# Description of the first marine-isolated member of the under-represented phylum *Gemmatimonadota*, and the environmental distribution and ecogenomics of *Gaopeijiales* ord. nov.

**DOI:** 10.1128/msystems.00535-24

**Published:** 2024-11-19

**Authors:** Yu-Qi Ye, Meng-Qi Ye, Xin-Yue Zhang, You-Zhi Huang, Zi-Yang Zhou, Yan-Jun Feng, Zong-Jun Du

**Affiliations:** 1 Marine College, Shandong University, Weihai, Shandong, China; 2 Shandong University-Weihai Research Institute of Industrial Technology, Weihai, Shandong, China; 3 SDU-ANU Joint Science College, Shandong University, Weihai, Shandong, China; 4 Shine-Dalgarno Centre for RNA Innovation, Division of Genome Science and Cancer, John Curtin School of Medical Research, Australian National University, Canberra, Australian Capital Territory, Australia; 5 State Key Laboratory of Microbial Technology, Shandong University, Qingdao, Shandong, China; Stellenbosch University, Stellenbosch, South Africa

**Keywords:** *Gemmatimonadota*, BD2-11 terrestrial group, *Gaopeijiales *ord. nov., marine bacteria, microbial ecology, taxonomy

## Abstract

The phylum *Gemmatimonadota* is widespread but rarely cultured and, in fact, there are only six described species isolated from soil, freshwater, and wastewater treatment. However, no isolates of *Gemmatimonadota* from marine environment have been described; thus, little is known about the physiology and metabolism of members of the marine lineages. In this study, four novel facultatively anaerobic bacterial strains belonging to *Gemmatimonadota* were isolated from marine sediments collected from Xiaoshi Island in Weihai, China, using an aerobic enrichment method. The integrated results of phylogenetic and phenotypic characteristics supported that these four strains represent one novel species in a novel genus, for which the name *Gaopeijia maritima* gen. nov., sp. nov. is proposed, as the first representative of novel taxa, *Gaopeijiales* ord. nov., *Gaopeijiaceae* fam. nov. in the class *Longimicrobiia. Gaopeijiales* was detected in 22,884 out of 95,549 amplicon data sets, mainly from soil. However, the highest mean relative abundances were in sponge (0.7%) and marine sediment (0.35%), showing salt-related character. Most of the *Gaopeijiales* subgroups potentially belong to the rare bacterial biosphere. The aerobic enrichment in this study could significantly increase the relative abundance of *Gaopeijiales* (from 0.37% to 2.6%). Furthermore, the metabolic capabilities inferred from high-quality representative *Gaopeijiales* genomes/MAGs suggest that this group primarily performs chemoorganoheterotrophic metabolism with facultatively anaerobic characteristics and possesses various secondary metabolite biosynthesis gene clusters (BGCs), mirroring those observed in the four novel strains.

**IMPORTANCE:**

Despite rapid advances in molecular and sequencing technologies, obtaining pure cultures remains a crucial research goal in microbiology, as it is essential for a deeper understanding of microbial metabolism. *Gemmatimonadota* is a widespread but rarely cultured bacterial phylum. Currently, there are only six cultured strains of this interesting group, all isolated from non-marine environments. Little is known about the physiology and metabolism of members of the marine lineages. Here we isolated and characterized four novel marine strains, and proposed a new order *Gaopeijiales* within *Gemmatimonadota*. Furthermore, the global distribution, environmental preference, and metabolic potential of *Gaopeijiales* are analyzed using public data. Our work enriches the resources available for the under-represented phylum *Gemmatimonadota* and provides insights into the physiological and metabolic characteristics of the marine lineage (*Gaopeijiales*) through culturology and omics.

## INTRODUCTION

The phylum *Gemmatimonadota*, one of the bacterial phyla established by molecular phylogenetic methods, was originally proposed according to environmental sequence data and named the BD/KS-B group (1–3). In 2003, the first pure culture (designated *Gemmatimonas aurantiaca*) belonging to the BD/KS-B group was isolated from enhanced biological phosphorus removal (EBPR) reactor samples, and the BD/KS-B group was proposed the novel bacterial phylum *Gemmatimonadota* (4).

Culture-independent methods indicated that *Gemmatimonadota* are ubiquitous residents of various environments and particularly common in soils, freshwater, sewage treatment reactors, and marine environments (5, 6). *Gemmatimonadota* members evolve as aerobic organotrophic species and exhibit diverse and flexible metabolisms across different environments. For instance, photoheterotrophy was predominantly found in freshwater, soda lakes, and wastewater, whereas CO oxidation was prevalent in soil, marine sediment, hydrothermal vents, host-associated, and groundwater environments (7). In addition, *Gemmatimonadota* exhibit different strategies for survival under phosphorus limitation in various environments (7). Prior works suggested that the most abundant phylogenetic groups of *nosZ*, involved in the reduction of N_2_O, are clade II *nosZ* genes affiliated with the *Gemmatimonadota* in the environment (8–10). And *nosZ* genes are present in *Gemmatimonadota* genomes from most environments, with exceptions noted in marine water and host-associated habitats (7). Anoxygenic phototrophic *Gemmatimonadota* were found in freshwater, wastewater, and soda lake sediment, particularly in euphotic zones of the freshwater lake, with different phototrophic groups evolving two independent photosystems (7, 11–13). In addition, research showed that *Gemmatimonadota* are likely involved in carbon fixation, especially in soda lakes, wastewater, and glacier environments (7, 11, 14, 15). Together, these studies suggest that the widely distributed *Gemmatimonadota* exhibit a diversity of metabolisms across various environments and likely play an essential role in ecological processes.

Based on the cultured species and environmental 16S rRNA gene sequences, *Gemmatimonadota* was divided into seven major lineages at the class level (*Gemmatimonadia*, *Longimicrobiia*, BD2-11 terrestrial group, PAUC43f marine benthic group, S0134 terrestrial group, AKAU4049, and MD2902-B12) in the SILVA database (release 138). The BD2-11 terrestrial group contains both soil source sequences and sequences from marine sediment and sponge (16). A recent study analyzed the global distribution and potential metabolism of the marine *Gemmatimonadota* group PAUC43f based on the 16S rRNA gene amplicon database and metagenome-assembled genomes (MAGs) (17). It proposed *Palauibacterales* as a new cosmopolitan thiamine-producing order, characterized by a saline-related nature and a chemoorganoheterotrophic and facultatively aerobic metabolism (17). However, no pure culture was isolated, *Palauibacterales* has not yet been approved by the International Committee on Systematics of Prokaryotes (ICSP).

Great advancements in sequencing technology have augmented our comprehension of the microbial realm and significantly broadened the “tree of life” (18–20). Nevertheless, obtaining pure culture is essential for a deep understanding of the physiological and metabolic characteristics of microorganisms. Unfortunately, the phylum *Gemmatimonadota* comprises only two validly published classes (*Gemmatimonadia* and *Longimicrobiia*) and six described cultured species (4, 21–25), according to the List of Prokaryotic names with Standing in Nomenclature (LPSN) (https://lpsn.dsmz.de/phylum/gemmatimonadota, accessed August 2024). Within the class *Gemmatimonadia*, five species from three genera (*Gemmatimonas*, *Gemmatirosa*, and *Roseisolibacter*) have been proposed, isolated from activated sludge (4), soil (21, 24), and freshwater environments (22, 25). *Longimicrobiia* consists of only one cultured member isolated from forest soil (23). However, none of the marine lineages within the *Gemmatimonadota*, as well as the BD2-11 terrestrial group, have been successfully isolated and taxonomically characterized up to now, and relatively little is known about their physiology and metabolism. This limitation greatly hinders research on *Gemmatimonadota*. Marine environments serve as a “seed bank,” where dormant bacteria are still present in the marine sediments but at a much lower abundance (26, 27). An enrichment system based on mixed culture, a broad method for mining microbial dark matter, could cultivate the “uncultured” microbes not only through enriching their abundance but also through the resuscitation mechanism (26). By applying enrichment techniques to marine samples, it may be possible to obtain the marine lineages within the *Gemmatimonadota*.

In this study, we isolated and described four novel *Gemmatimonadota* bacterial strains from marine sediments using an aerobic enrichment method. Through polyphasic taxonomy, we propose the establishment of new taxa, *Gaopeijia maritima* gen. nov., sp. nov., and *Gaopeijiales* ord. nov., *Gaopeijiaceae* fam. nov. in the class *Longimicrobiia*. We predict that the members of *Gaopeijiales* from various environments may exhibit different metabolic potentials. To study the geographical distribution and environmental preference of *Gaopeijiales*, we conducted an extensive search in 95,549 publicly available 16S rRNA gene amplicon data sets from the Sequence Read Archive (SRA). Furthermore, functional gene annotation and metabolic pathway reconstruction using high-quality representative genomes/MAGs were performed for the purpose of ecogenomic characterization. Our work provides insights into the physiology, metabolism, and ecology of marine *Gemmatimonadota* through both culturology and omics.

## RESULTS AND DISCUSSION

### Isolation, cultivation, and 16S rRNA gene similarity analysis of novel *Gemmatimonadota* members

Strains DH-78^T^, DH-20, CCK-12, and Y43 were isolated from the aerobic enrichment system of marine sediments and subsequently cultured on marine agar 2216 (MA; Becton Dickinson). The complete 16S rRNA gene sequences of the four strains (1,563 bp) were obtained and aligned with public databases. Results revealed that these isolates exhibited similarity values ranging from 83.2% to 86.2% with cultured members of *Gemmatimonadota*, with the highest similarity observed with *Longimicrobium terrae* CB-286315^T^ (85.9%–86.2%) (Table S1). While the 16S rRNA gene sequences of these four strains exhibited very high similarity to each other (99.2%–100%), their BOX-PCR profiles (Fig. S1) tentatively indicated that they constituted a genetically distinct and heterogeneous group rather than being clones of a single strain.

### Morphology, phenotype, and chemotaxonomic properties

Cells of the isolates DH-78^T^, DH-20, CCK-12, and Y43 were Gram-stain-negative, short rod-shaped, with widths of 0.4–0.6 µm and lengths of 0.7–4.5 µm (Fig. S2). Growth was observed under aerobic and microaerophilic (8%–9% O_2_) conditions, but not under anaerobic conditions. Optimum growth of the four strains was observed at 35–37°C. The temperature range of growth exceeded those of the related type strains (22, 23) (Table 1). In addition, all four isolates required at least 1% (wt*/*vol) NaCl for growth, with the optimum NaCl concentration being 3% (wt/vol). Notably, the maximum NaCl concentration tolerated by these four marine isolates was 7%–8% (wt/vol), which significantly surpassed that of the six isolates from non-marine environments previously reported in *Gemmatimonadota* (4, 21–25).

**TABLE 1:** Comparison of phenotypic characteristics of strains DH-78^T^, DH-20, CCK-12, Y43, and related type strains^*a*^

Characteristics	1	2	3	4	5^*b*^	6^*c*^
Isolation	Intertidal sediments	Intertidal sediments	Intertidal sediments	Intertidal sediments	Forest soil	Wastewater reactor
Method	Aerobic enrichment	Aerobic enrichment	Aerobic enrichment	Aerobic enrichment	Diffusion sandwich	NM-1 medium
Colony color	Pink	Pink	Pink	Salmon color	Pale salmon color	Faintly orange to pink
Cell shape	Oval or short rods	Oval or short rods	Oval or short rods	Oval or short rods	Short to long rods	Short rods
Cell size (μm)	0.4–0.6 × 0.7–4.5	0.4–0.6 × 0.7–4.5	0.4–0.6 × 0.7–4.5	0.4–0.6 × 0.7–4.5	0.4–0.6 × 1.3–15	0.7 × 2.5–3.2
Temperature range (°C)	20–45	20–45	20–45	20–45	10–33	16–30^*d*^
NaCl range (%, wt/vol)	1–8	1–8	1–7	1–8	0–0.4	0–0.8^*e*^
Optimum pH	7.5	8.0–8.5	7.5	8.0	7.0–7.5	7.0
Voges–Proskauer reaction	−	−	−	+	−	nd
Hydrolysis of cellulose	−	−	−	−	+	nd
Enzyme activities:						
Gelatinase	−	w	+	+	+	v^*b*^
Esterase lipase (C8)	+	w	+	w	+	+^*b*^
Valine arylamidase	+	+	+	+	−	+^*b*^
Cystine arylamidase	w	+	+	+	−	w^*b*^
α-chymotrypsin	+	+	+	+	−	+^*b*^
N-acetyl-β-glucosaminidase	−	−	−	−	−	+^*b*^
Oxidation of:						
d-turanose	w	w	w	−	nd	−
α-d-lactose	−	−	−	−	+	−
l-rhamnose	w	w	w	−	+	−
l-histidine	+	+	+	+	−	−
Major quinone	MK-6	MK-6	MK-6	MK-6	MK-8	MK-9

^*a*^ Strains: 1, DH-78^T^; 2, DH-20; 3, CCK-12; 4, Y43; 5, *Longimicrobium terrae* CB-286315^T^; 6, *Gemmatimonas aurantiaca* T-27^T^. All data were from this study unless indicated otherwise. +, positive; −, negative; w, weakly positive; v, variable response; nd, not detected/no data.

^*b*^ Data from Pascual et al. (23).

^*c*^ Data were compiled from previous studies (4, 22, 23).

^*d*^ Reported as 16–30°C by Zeng et al. (22).

^*e*^ Reported as 0%–0.8% (wt/vol) NaCl by Zeng et al. (22).

Some or all of the four strains demonstrated the ability to hydrolyze Tween 20 (all strains were positive), Tween 40 (strains CCK-12 and Y43 were weakly positive), and Tween 60 (strains DH-78^T^ and DH-20 were weakly positive), indicating their potential to utilize certain polyether polyols for growth. None of them exhibited hydrolytic activity toward Tween 80, starch, cellulose, alginate, casein, or agar. However, they all exhibited positive reactions for d-fructose-6-PO4, d-galacturonic acid, l-galactonic acid lactone, d-glucuronic acid, and glucuronamide, with weak positivity for d-fructose, d-fucose, and l-fucose. In addition, in the API 50CH strip, all strains produced acids from d-ribose, d-fructose, l-sorbose, d-tagatose, esculin ferric citrate, d-lyxose, l-xylose, and potassium 5-ketogluconate. These combined results suggest that the novel strains primarily metabolize monosaccharides and derivatives, rather than complex compounds like polysaccharides. The main distinguishing characteristics of the four novel strains compared to related type strains are summarized in Table 1. These four strains differed in multiple phenotypes, further suggesting that they represent distinct strains.

The major fatty acids of the four novel isolates were iso-C_15:0_ (31.6%–43.4%), C_16:1_ *ω*5*c* (13.2%–16.6%), and iso-C_14:0_ (9.7%–12.2%), which differed significantly from the nearest known type strains, *Longimicrobium terrae* CB-286315^T^ and *Gemmatimonas aurantiaca* T-27^T^ (Table S2). Unlike the predominant respiratory quinone (MK-8 or MK-9) observed in reported *Gemmatimonadota* isolates (4, 21–25), the primary respiratory quinone of the four strains in this study was MK-6. The major polar lipids detected in these four strains were phosphatidylethanolamine (PE), phosphatidylglycerol (PG), diphosphatidylglycerol (DPG), and aminolipid (AL) (Fig. S3).

### Phylogenetics based on 16S rRNA gene

**Fig 1:**
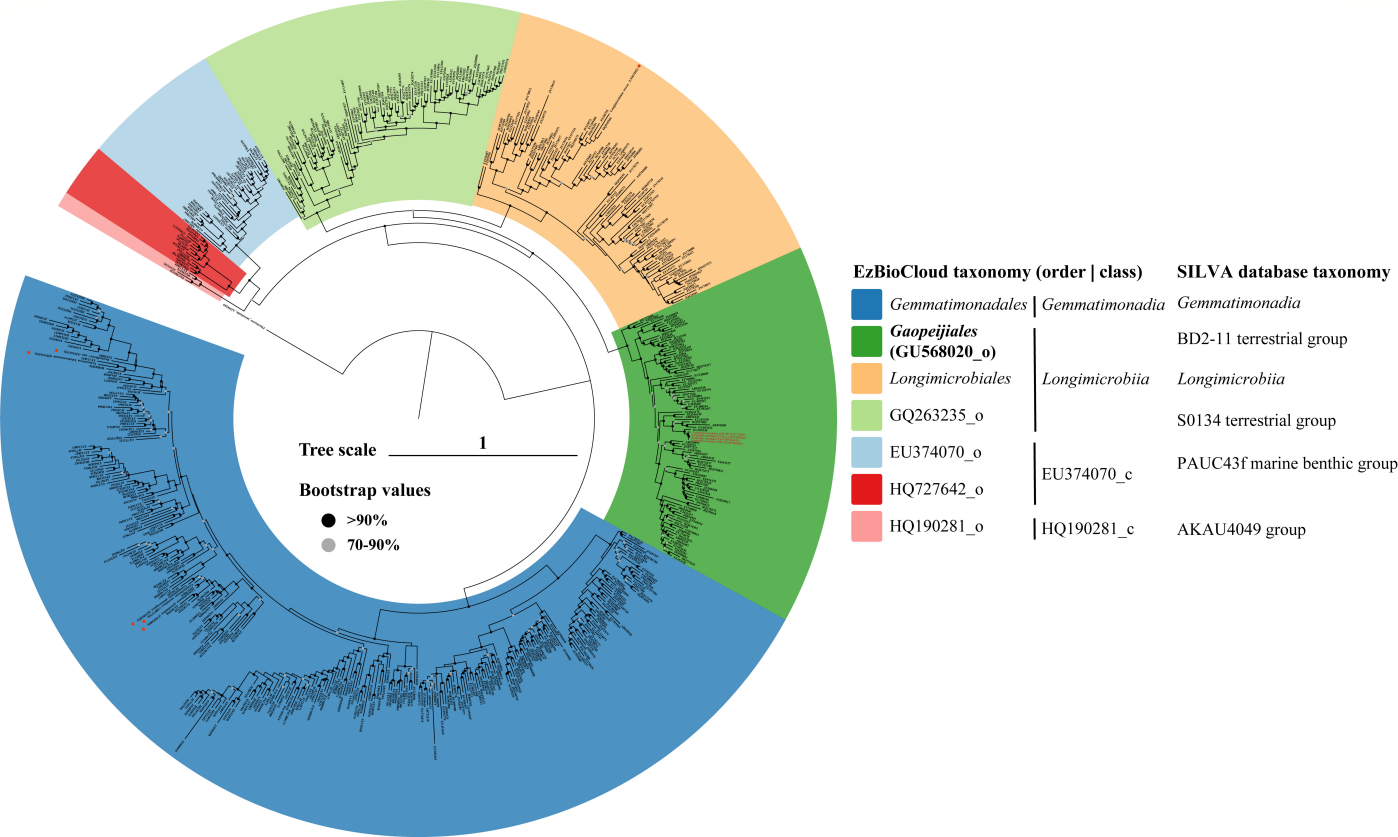
The maximum-likelihood phylogenetic tree based on 655 high-quality representative 16S rRNA gene sequences (more than 1,200 bp), including cultured representatives and environmental clones. *Fibrobacter intestinalis* NR9^T^ was used as the outgroup. Bootstrap values above 70% (1,000 replicates) are shown at branch nodes. Accession numbers are indicated using leaf information on the phylogenetic tree.

**Fig 2:**
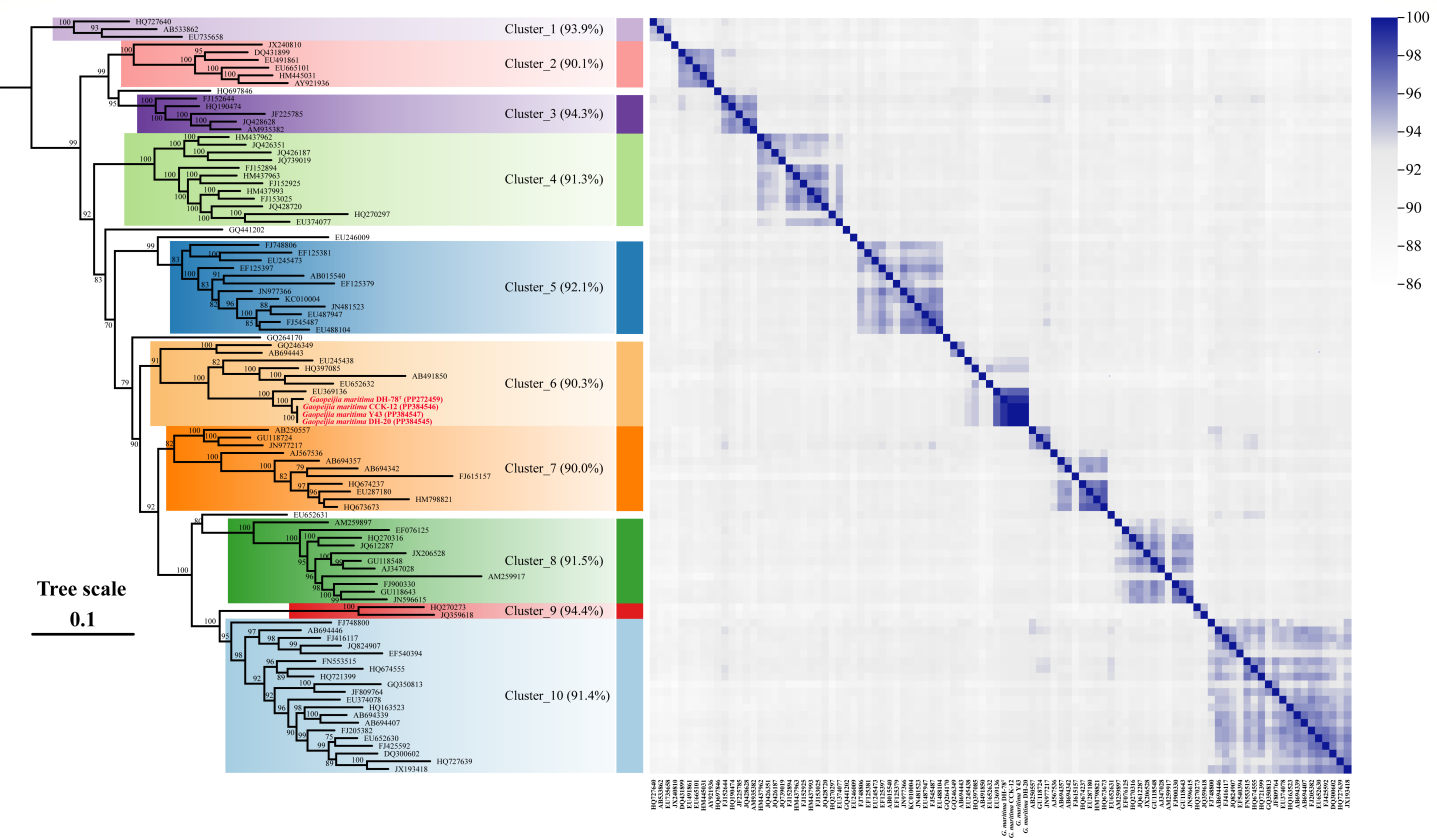
Taxonomic classification of *Gaopeijiales* based on phylogeny and 16S rRNA gene sequence similarity. The left phylogenetic tree was reconstructed based on 655 high-quality representative 16S rRNA gene sequences and displays only the branches of *Gaopeijiales. Fibrobacter intestinalis* NR9^T^ was used as the outgroup. Bootstrap values above 70% (1,000 replicates) are shown at branch nodes. The right heat map illustrates the 16S rRNA gene sequence similarity values. *Gaopeijiales* is divided into 10 subgroups.

The maximum-likelihood phylogenetic tree based on 16S rRNA gene sequences revealed that the novel isolates, along with several uncultured clones, formed a separate branch affiliated with the BD2-11 terrestrial group with high bootstrap values (Fig. S4). The robust phylogenetic tree based on 655 high-quality 16S rRNA gene sequences (Table S3) showed that the four strains were clustered into an unclassified order (GU568020) within the class *Longimicrobiia* (Fig. 1). On the whole, the two phylogenetic trees have similar topology, the order GU568020 corresponds to the BD2-11 terrestrial group. The above results suggest that the four strains most likely represent a novel species of a novel order in the class *Longimicrobiia* of *Gemmatimonadota*. Moreover, the unclassified order formed 10 sequence clusters, which was supported by both FastTree (Fig. S5) and IQ-TREE algorithm (Fig. 2). The novel species in this study belonged to cluster 6. Generally, there were high intra-cluster diversities, with the majority of clusters holding ≤94.0% intra-group minimum similarities that ranged from 90.0% to 94.4% (Fig. 2).

### Genome features and phylogenomics

A total of 1.13 Gbp second-generation sequencing raw bases and 0.33 Gbp third-generation sequencing data were obtained, resulting in a complete genome of strain DH-78^T^. The genome of strain DH-78^T^ was represented by one circular chromosome with a total length of 4,224,903 bp and a G + C content of 70.4% (Table S4). The NCBI Prokaryotic Genome Annotation Pipeline (PGAP) results predicted the presence of 3,586 protein-coding genes and 53 RNA genes (1 16S rRNA, 2 5S rRNA, 2 23S rRNA, 2 ncRNA, and 46 tRNA genes). The genome size of the four novel isolates ranged from 4.20 to 4.25 Mbp, which was less than that of the six effectively published *Gemmatimonadota* members (Table S4).

**Fig 3:**
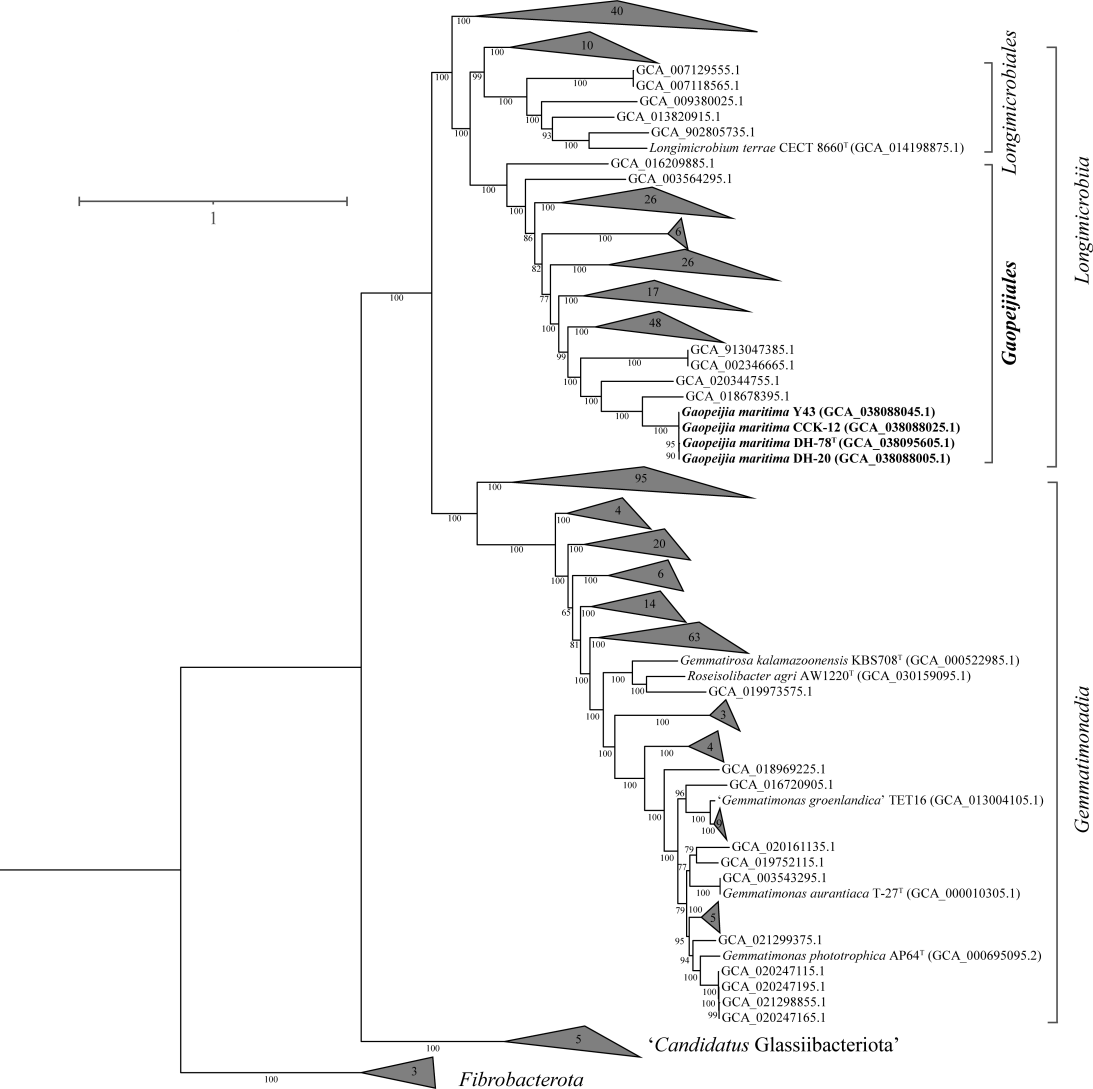
The maximum-likelihood phylogenetic tree (based on 120 ubiquitous single-copy proteins in GTDB) of 436 genomes. Three genomes affiliated with the phylum *Fibrobacterota*, *Fibrobacter intestinalis* (GCA_900142455.1), *Fibrobacter succinogenes* (GCA_000146505.1), and *Fibrobacter elongatus* (GCA_003149165.1), were used as the outgroup. Bootstrap values (1,000 replicates) are shown at branch nodes.

The average amino acid identity (AAI) and the percentage of conserved proteins (POCP) values computed between the four novel strains and cultured *Gemmatimonadota* members ranged from 53.4% to 56.1% and from 24.8% to 29.2%, respectively (Fig. S6), which were far below the delineations of species and genus (28, 29). Furthermore, a total of 429 high-quality genomes/MAGs belonging to *Gemmatimonadota* downloaded from the Genome Taxonomy Database (GTDB) were used for reconstructing the phylogenomic tree (Table S5). The maximum-likelihood phylogenetic tree based on 120 ubiquitous single-copy proteins showed that the four strains and some uncultured clones formed a separate branch in the class *Longimicrobiia* with high bootstrap values, which supported the conclusion they represent a novel order (Fig. 3).

### Environmental distribution of *Gaopeijiales* ord. nov.

**Fig 4:**
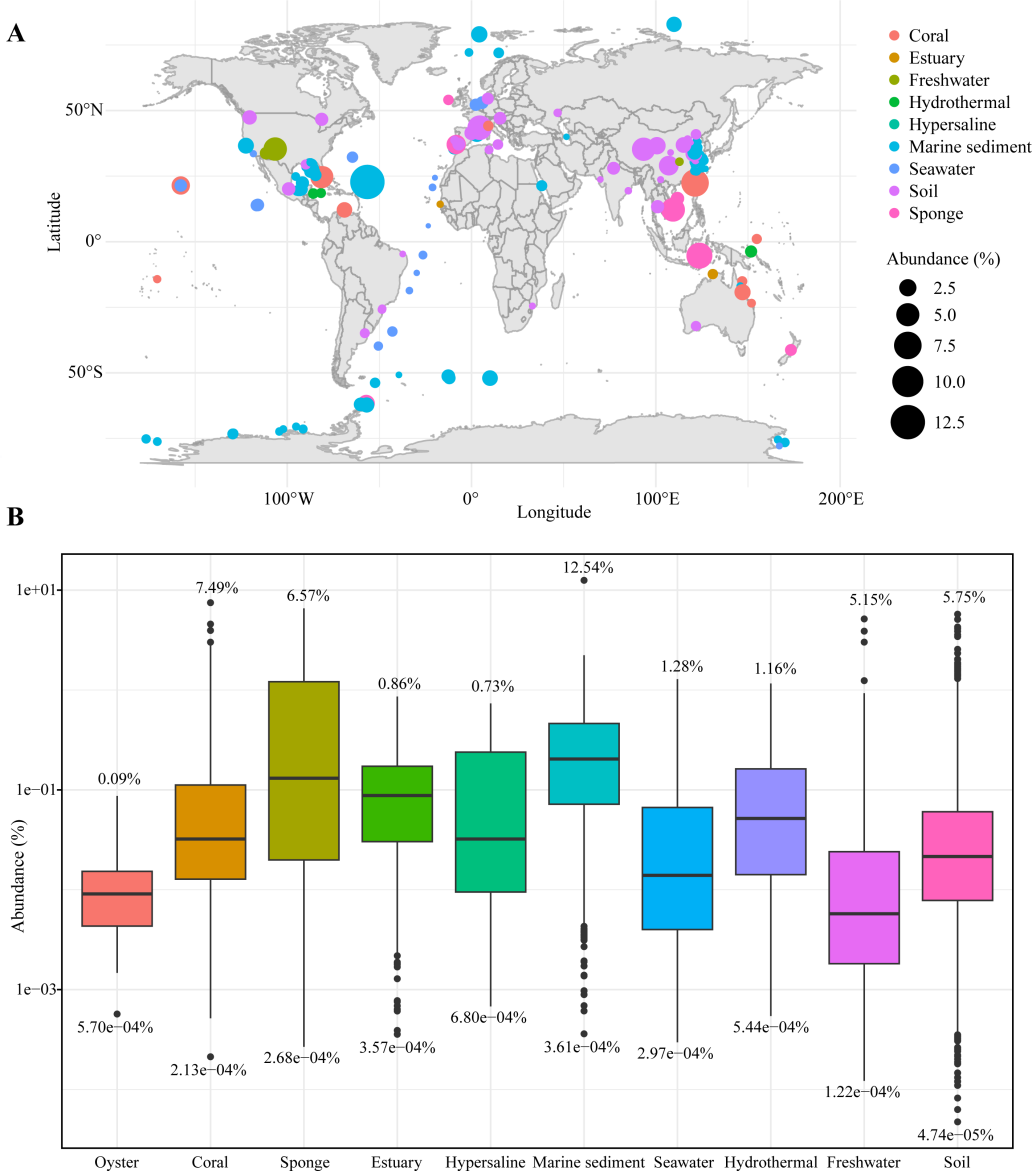
The environmental distribution and relative abundance of *Gaopeijiales* based on 16S rRNA gene sequences. **(A)** Worldwide distribution and environments where *Gaopeijiales* sequences were detected with a relative abundance above 0.5%. **(B)** The boxplot, presented in logarithmic scale, illustrates the relative abundances of *Gaopeijiales* across various environments, quantified as the percentage of *Gaopeijiales* 16S rRNA gene sequences in relation to the total sequence count (*P*-values of pairwise Wilcoxon tests are shown in Table S7). Values above and below each boxplot indicate the maximum and minimum abundance, respectively, in the corresponding environment. Number of data sets per environment: oyster, *n* = 41; coral, *n* = 814; sponge, *n* = 245; estuary, *n* = 272; hypersaline, *n* = 8; marine sediment, *n* = 2,018; seawater, *n* = 1,052; hydrothermal, *n* = 102; freshwater, *n* = 1,389; soil, *n* = 16,943.

The *Gaopeijiales* members were detected around the world in various environments including marine habitats (such as coral, sponge, estuary, marine sediment, seawater, and hydrothermal vent), hypersaline lake, freshwater, and soil (Fig. 4A). The percentages of reads belonging to *Gaopeijiales* to total reads in different environmental samples were calculated to estimate the relative abundance for more insights into environmental distribution (Fig. 4B). *Gaopeijiales* 16S rRNA gene sequences were detected in 22,884 of the 95,549 amplicon data sets analyzed, mainly from soil environment (Table S6). However, the highest mean relative abundances were in marine environments (except oysters) and hypersaline lakes, especially marine sediment and sponges (Table S7).

**Fig 5:**
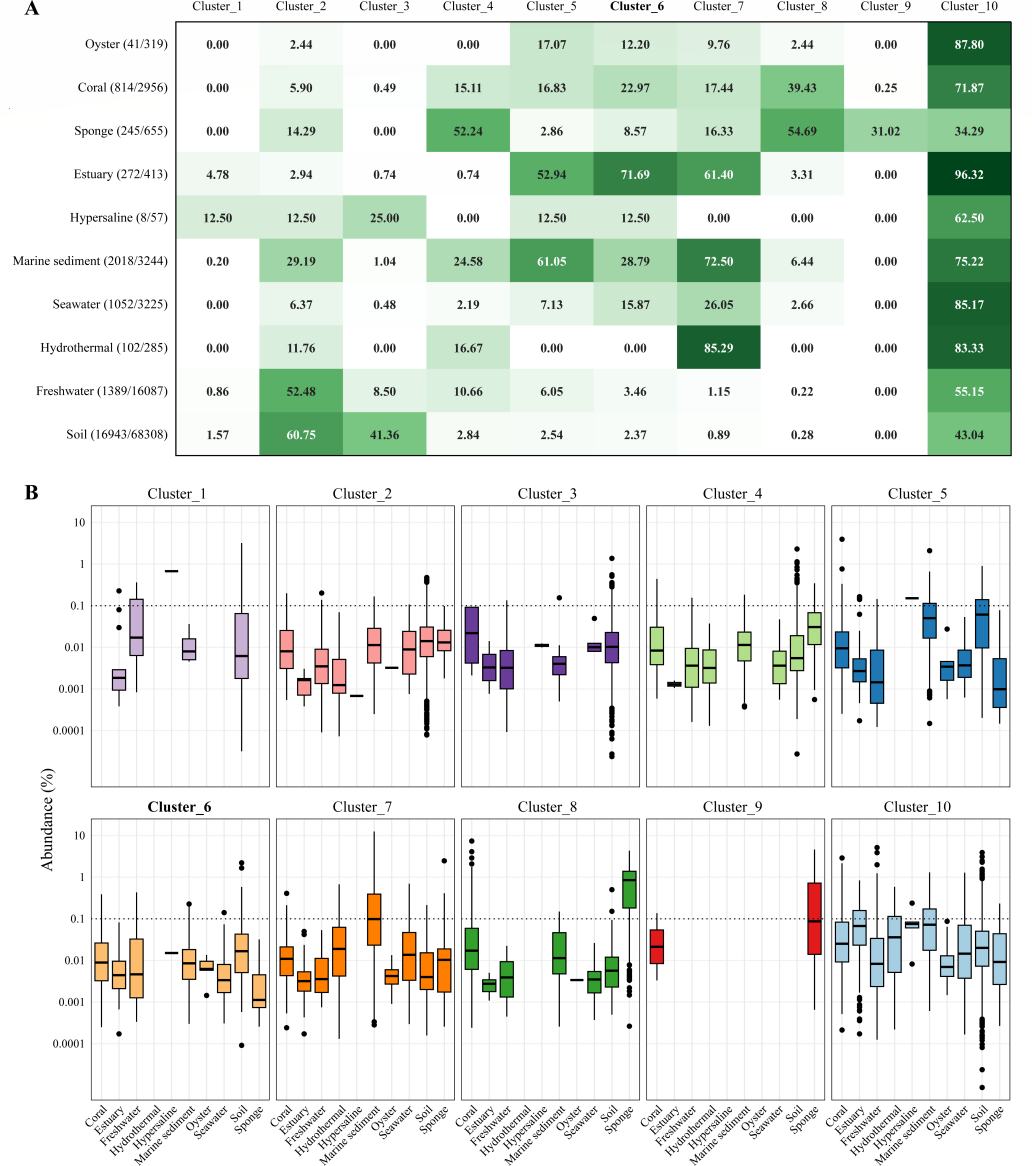
The environmental distribution of 10 subgroups affiliated with *Gaopeijiales*. **(A)** The percentage of samples per environment in which each subgroup is detected with respect to the total number of samples where *Gaopeijiales* is present. The highest values are depicted in green and the lowest in white. The values in parentheses to the right of the environment type represent the number of samples where *Gaopeijiales* subgroups were detected out of the total number of samples analyzed for that environment. The novel species characterized in this study belongs to the cluster 6 (in bold). **(B) **The relative abundance of each subgroup in each environment quantified as the percentage of *Gaopeijiales* 16S rRNA gene sequences in relation to the total number of sequences. The horizontal dashed line represents a relative abundance of 0.1%, as a threshold for rare biospheres (˂ 0.1%). *P*-values of pairwise Wilcoxon tests are shown in Table S7.

According to the topological structure of the phylogenetic tree and 16S rRNA gene similarity, the order *Gaopeijiales* was divided into 10 clusters (Fig. 2). To analyze the environmental distribution and habitat preferences of these subgroups, their frequencies and abundances in different habitats were calculated (Fig. 5). The results in Fig. 5A showed that the detection frequency of each subgroup differed across environments. Clusters 2, 4, 5, 6, 7, and 10 exhibited a broad environmental distribution, while some clusters were only detected in limited samples from specific habitats. For instance, cluster 1 was mainly found in hypersaline samples, and cluster 9 was only from host-associated environments (sponge and coral). Only clusters 2, 4, 7, and 10 were found in hydrothermal vent samples, showing evolutionary branching differences in various environments. Furthermore, the abundance of 10 clusters in different habitats, as shown in Fig. 5B, indicated that most of the *Gaopeijiales* subgroups potentially belong to the rare bacterial biosphere, principally contributing to the overall biodiversity despite their low abundance of less than 0.1% (30, 31). The mean relative abundances of certain subgroups in particular environments exceeded 0.1%. For instance, cluster 7 exhibited significant prevalence in marine sediment compared to other environments, whereas cluster 9 demonstrated notably higher abundance in sponge habitats than in others (Table S7). These findings imply that certain *Gaopeijiales* subgroups may be indigenous inhabitants of microbiomes within distinct habitats.

### Relative abundance of *Gaopeijiales* ord. nov. in the enrichment system

The high-throughput sequencing targeting the 16S rRNA gene of 20 aerobic enrichment samples was performed and 982,574 sequences were obtained. There were 640,220 sequences clustered into 4,858 OTUs after sequence normalization, and 43 OTUs were assigned to *Gemmatimonadota* (Table S8). The phylogenetic tree containing *Gemmatimonadota* OTUs (Fig. S7) showed that 32 OTUs were identified as BD2-11 terrestrial group, which was consistent with the results of amplicon sequencing (Table S8). Therefore, these 32 OTUs were used to analyze changes in the relative abundance of the novel taxon during enrichment. The results showed that the average relative abundance of *Gaopeijiales* in the original environment (marine sediment) on 0 day was 0.37%, which was consistent with the environmental distribution analysis above (0.35%, Table S7). Noteworthy, the relative abundance of *Gaopeijiales* decreased in the early enrichment period (0–5 d), and gradually increased to 2.6% at 30 days (Fig. S8). It is suggested that aerobic enrichment can significantly increase the relative abundance of this novel group and is a potentially effective method to explore *Gemmatimonadota* bacteria.

### Genome metabolic potential of *Gaopeijiales* ord. nov.

**Fig 6:**
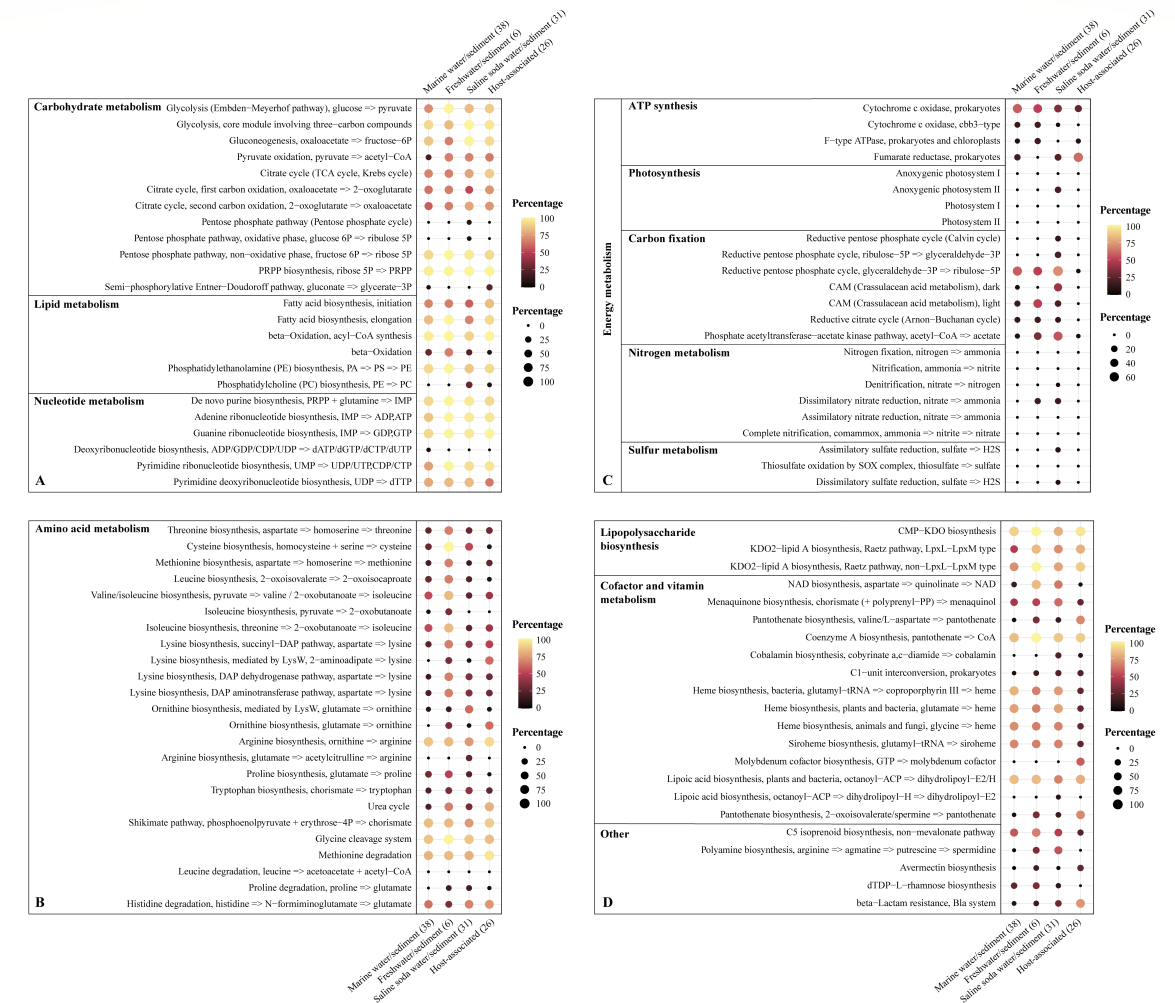
The bubble plot showing the percentage of genomes containing complete or almost complete metabolic modules in various environments. Dot color and size indicate the percentage values in any given environment, with the darkest color and smallest size of the dot marking the lowest value. The number of genomes in each environment is indicated in the parenthesis. The details about metabolic modules and key genes presence/absence are shown in Tables S9, S10, and S11.

**Fig 7:**
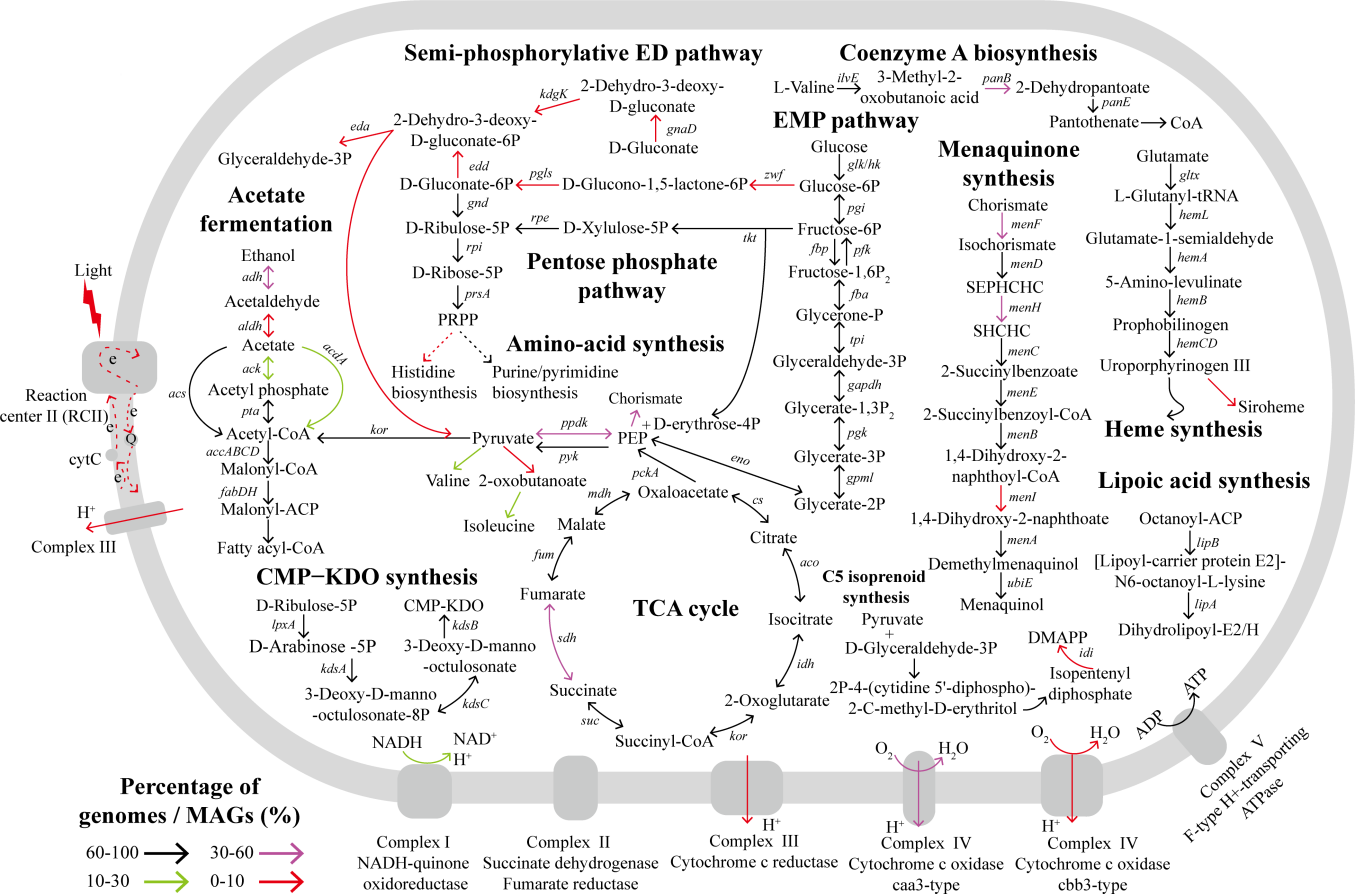
Metabolic reconstruction of *Gaopeijiales* showing some of the key pathways. The color of the arrow represents the proportion of genomes containing the corresponding gene or capable of performing the metabolic reaction. The italicized letters near the arrows in the figure indicate the genes involved in the corresponding pathway. The details about metabolic pathways and key genes presence/absence are shown in Tables S9, S10, and S11.

The metabolic pathways and clusters of orthologous groups (COG) were first analyzed between the four novel isolates and cultured members of *Gemmatimonadota* (Fig. S9 and S10). The results indicated that they had major differences in carbohydrate, energy, and amino acid metabolisms (Fig. S9), and had a higher number of genes encoding amino acid metabolism than carbohydrate metabolism (Fig. S10), pointing to the great importance of amino acid metabolism in *Gemmatimonadota*. To further enlighten the metabolic characteristics and ecological role of *Gaopeijiales*, the metabolic capabilities and functional gene analysis based on 101 non-redundant *Gaopeijiales* genomes/MAGs from different habitats were performed (Fig. 6 and 7; Tables S9 to S11). The numbers of dereplicated genomes within each category were as follows: marine water/sediment 38, freshwater/sediment 6, saline soda water/sediment 31, and host associated 26 (Table S5).

#### Central carbon metabolism

Concerning central carbon metabolism, complete or almost complete glycolysis and gluconeogenesis were found in most members from natural and host-related environments, like the first described species of *Gaopeijiales* in this study (Fig. 6A and 7; Fig. S9), suggesting that *Gaopeijiales* was a chemoorganotrophic group and may adapt to growing conditions where carbon sources are absent or limited by converting non-sugar substances. In low-nutrient marine environments, the four novel strains could degrade soluble organic matter through glycolysis, thereby supporting their survival and releasing intermediate metabolites that serve as nutrients for other microorganisms, which may influence the carbon and nutrient cycles in the ocean. In the analyzed environments, approximately 66.7%–100% of *Gaopeijiales* members had complete or nearly complete gluconeogenesis (Fig. 6A; Table S9), indicating that this process, which involves storing sufficient energy in glucose followed by the synthesis of complex compounds, should be prevalent in *Gaopeijiales*. However, the previously reported *Gemmatimonadota* strains could not perform complete glycolysis and gluconeogenesis (Fig. S9) due to the absence of the fructose-bisphosphate aldolase gene (*fba*). It was noticed that almost all of analyzed genomes were missing the genes encoding glucose-6-phosphate dehydrogenase (G6pd, KEGG Orthology K00036), 6-phosphogluconolactonase (Pgl, K07404), and phosphogluconate dehydratase (Edd, K01690), which are key enzymes in Entner-Doudoroff (ED) pathway (KEGG Module M00008) (Table S10). It suggests that acquiring pyruvate through the ED pathway as an alternative may not be common in *Gaopeijiales*, which is consistent with previous findings (13). The genes encoding citrate synthase (Cs, K01647), aconitase (Aco, K01681), and isocitrate dehydrogenase (Idh, K00031), involved in the first carbon oxidation step of citric acid cycle (M00010), were detected in the majority of *Gaopeijiales* genomes (with averages of 93.1%, 83.2%, and 78.2%, respectively) in the analyzed environments (Table S10). However, the genes responsible for encoding α-ketoglutarate dehydrogenase complex (OGDC) were absent in over half of genomes from saline soda water/sediment, and genes encoding succinate dehydrogenase (Sdh, K00239−K00242) were observed in less than 20% of the genomes in marine and freshwater environments (Table S10). The pentose phosphate pathway (PPP), including the oxidation and non-oxidation phase, provides an alternative to glycolysis for glucose oxidation (13). Only two genomes from saline soda water/sediment of the analyzed genomes had a complete oxidation phase (M00006) (Table S9). Genes for the conversion of phosphorylated carbohydrates in the nonoxidative phase (M00007) are present in over 90% of the *Gaopeijiales* genomes (Fig. 6A; Table S10). The ribose produced by this phase is one of the important precursors required for the synthesis of DNA and RNA, supporting nucleic acid synthesis in cells.

#### Lipid metabolism

Lipid metabolism involves the breakdown, synthesis, and storage of lipids, with fatty acids playing a crucial role in this process. The initiation and elongation steps of fatty acid synthesis were observed in the majority of *Gaopeijiales* members (Fig. 6A). Moreover, the four novel strains in this study exhibited esterase (C4), esterase lipase (C8), and phosphatase activities, facilitating the acquisition of fatty acids through lipolysis. β-oxidation is the mitochondrial aerobic process of breaking down a fatty acid into acetyl-CoA units and is the major metabolic pathway by which energy is released from fatty acids (32). Acyl-CoA synthetase (ACSL, K01897), converting fatty acids into acyl−CoA (M00086), was annotated in almost all *Gaopeijiales* genomes (more than 90%) in various environments (Table S9). However, complete or almost complete β-oxidation pathway was found in marine water/sediment (28.9%), freshwater/sediment (66.7%), saline soda water/sediment (22.6%), and host associated (7.7%), showing a difference (Fig. 6A). Phosphatidylcholine (PC), a typical eukaryotic membrane phospholipid, is estimated to be present in about 10%–15% of all bacteria (33, 34). The methylation pathway and phosphatidylcholine synthase pathway are two known approaches synthesizing PC in bacteria. PC produced by phosphatidylethanolamine (PE) methylation pathway was found in soda water/sediment (29.0%) and host-associated habitat (7.7%) (Fig. 6A). PC may enhance the resistance of these bacteria to hypo- and hyperosmotic environmental conditions and play an important role in the interactions between symbiotic bacteria and their eukaryotic hosts (34, 35).

#### Amino acid metabolism

Amino acids play important roles in bacterial nutrition and growth as essential energy sources and fundamental building blocks of the proteome. Although certain amino acid syntheses remained complete in most genomes from a particular habitat, the majority of *Gaopeijiales* members across various environments exhibited amino acid auxotrophies (Fig. 6B and 7). Ornithine can be synthesized from glutamate through the N-Acetyl-l-glutamate pathway and the LysW-l-glutamate pathway. In saline soda water/sediment, over half of the members had the N-Acetyl-l-glutamate pathway, whereas the proportion in other environments was less than 20% (Fig. 6B). The members of *Gaopeijiales* in host-associated habitats appeared to prefer synthesizing ornithine through the LysW-l-glutamate pathway (Fig. 6B). Moreover, the synthetic pathway from glutamate to arginine appeared to be unique to the members of saline soda environments (Fig. 6B). In marine, saline and host-associated environments, the most common putative auxotrophies were observed for threonine, methionine, leucine, and proline (Fig. 6B). However, under certain conditions, the auxotrophies have a selective advantage due to the corresponding reduction in the energy costs required for synthesis, and the amino acid cross-feeding can be an ecologically stable strategy when interacting partners complement each other in their metabolic capabilities (36). Interestingly, the four novel strains isolated in this study possessed the most pathways for amino acid synthesis (Fig. S9), such as threonine, leucine, proline, and histidine. The accumulation of amino acids may enable these marine strains to adapt to the high osmotic pressure of marine environments (37), and these prototrophies may play a relevant ecological role in providing amino acids to the marine community (17). The successful isolation of prototrophic strains suggests the potential importance of either endogenous synthesis or exogenous addition of certain amino acids for the isolation and culture of *Gaopeijiales*. The shikimate pathway is crucial for the biosynthesis of aromatic amino acids in plants and bacteria, and its intermediates, including dehydroshikimic acid (DHS), shikimate (SA), and chorismate (CHA), serve as essential precursors for synthesizing numerous high-value natural products, such as phenylpropanoids and flavonoids (38, 39). Notably, the shikimate pathway was present in about 80% of the *Gaopeijiales* genomes analyzed (Fig. 6B) and was complete in the four isolates in this study (Fig. S9), indicating they could be valuable in medicine and biotechnology. Regarding amino acid degradation, glycine cleavage system, methionine degradation, and histidine degradation were prevalent in the *Gaopeijiales* members (Fig. 6B). Biochemical investigations in this study revealed that the four novel strains were capable of oxidizing l-histidine as the sole carbon source, suggesting their ability to thrive by deriving energy from amino acid oxidation (Table 1).

#### Energy metabolism

In terms of energy metabolism, less than 20% of members from saline soda water/sediment were found to possess anoxygenic photosystem II (Fig. 6C) and genes encoding the type I large subunit of ribulose-1,5-bisphosphate carboxylase/oxygenase (RuBisCO, K01601) (Table S11). It suggests *Gaopeijiales* gains energy primarily through oxidative phosphorylation coupled with the redox of organic matter, rather than through photosystem and inorganic carbon, which was supported by the four chemoheterotrophic strains in this study. NADH-quinone oxidoreductase (complex I) encoded by *nuo* is the largest and most complicated energy-transducing enzyme complex in both bacterial and mitochondrial aerobic respiratory chains (40–42). The four novel strains lacked NADH-quinone oxidoreductase but possessed Na^+^-transporting NADH-ubiquinone oxidoreductase encoded by *nqr*, which couples the exergonic oxidation of NADH to the transport of Na+ across the cytoplasmic membrane (43). Cytochrome c oxidase (complex IV) in *Gaopeijiales* were mainly *cox*-encoded caa3-type and *cco*-encoded cbb3-type (Table S11). The caa3-type predominated across all analyzed environments, while the cbb3-type was primarily found in marine and freshwater environments, albeit in smaller proportions (Fig. 6C; Table S11). The cbb3-type cytochrome c oxidase usually exhibits high affinities for O_2_ (44), suggesting that some marine and freshwater inhabitants of *Gaopeijiales* may use oxygen at low concentrations for aerobic respiration. Furthermore, anaerobic fermentation was observed in *Gaopeijiales*. For instance, over 60% of host-related inhabitants had fumarate reductase (Fig. 6C) (7), an enzyme that reduces fumaric acid to succinate by releasing energy in the absence of oxygen (45). Phosphate acetyltransferase-acetate kinase pathway, another anaerobic fermentation process, was also found in *Gaopeijiales*, including the novel isolates in this study (Fig. 6C; Fig. S9). They could utilize certain simple sugars for anaerobic fermentation (Table 1). These results suggest that *Gaopeijiales* presents a chemoorganoheterotrophic and facultatively anaerobic metabolism, like the order *Palauibacterales* of *Gemmatimonadota* (17).

Most members of *Gaopeijiales* were unable to carry out complete nitrogen metabolism, such as nitrogen fixation, nitrification, and denitrification (Fig. 6C). Only a small portion of genomes from saline soda water/sediment exhibited potential denitrification and dissimilatory reduction capabilities (Fig. 6C), which is consistent with previous findings that certain *Gemmatimonadota* inhabitants in soda lakes have the potential for dissimilatory nitrate reduction (7, 11). However, this does not imply a complete absence of local metabolism. In spatially structured communities, local exchange among cooperative bacteria increases reciprocity (46, 47). The prior studies revealed that *Gemmatimonadota* is one of the potential groups performing N_2_O reduction (8, 9) and demonstrated that N_2_O can help sustain the viability of *Gemmatimonas aurantiaca* T-27 ^T^ during temporary anoxia, although N_2_O reduction was not coupled to growth (10, 48, 49). Nevertheless, nitrous-oxide reductase (NosZ, K00376) was uncommon in *Gaopeijiales* (0%–32.3%, Table S10). Instead, the NO-forming nitrite reductase gene (*nirK*) was detected in all analyzed environments (7). In this study, genes encoding nitrite reductase (NirK, K00368; NirS, K15864) were found mainly in marine water/sediment, including the novel strains (Table S10). As for sulfur metabolism, *Gemmatimonadota* exhibits a relatively straightforward process (Fig. 6C). Assimilatory sulfate reduction was found mainly in soil and soda lake members, and a complete thiosulfate oxidation pathway *via* the SOX system was not observed in any *Gemmatimonadota* genomes, although they contained some genes (7, 11).

#### Lipopolysaccharide, cofactor and vitamin biosynthesis

Lipopolysaccharide (LPS) is a major component of the outer membrane in Gram-negative bacteria (50). CMP-3-deoxy-d-manno-octulosonate (CMP-KDO) and KDO2-lipid A biosynthesis were a common feature for marine, freshwater/sediment, saline soda water/sediment, and host-associated genomes (Fig. 6D and 7). Nicotinamide adenine dinucleotide (NAD) is an essential cofactor in living systems, involved in bacterial stress response and resistance to phages (51–53). The complete and nearly complete NAD biosynthesis was found in most genomes from freshwater/sediment (83.3%) and saline water/sediment (64.5%), while it was only sporadically present in other analyzed environments (Fig. 6D). Molybdenum cofactor (Moco) forms part of the active centers of all molybdenum enzymes in living organisms (54, 55). However, a significant number of bacteria and unicellular eukaryotes do not need molybdenum, whereas all multicellular eukaryotes are dependent on Moco (56–58). Only members of *Gaopeijiales* from host-associated habitats (mainly sponges) exhibited the potential for Moco biosynthesis (Fig. 6D), which indicates that symbionts may synthesize Moco to share with host sponges. Coenzyme A, an essential cofactor in metabolism, could be synthesized by most *Gaopeijiales* inhabitants in various environments (Fig. 6D and 7) (7). Furthermore, the presence of multiple pathways for heme synthesis appeared to be a shared characteristic among free-living members of *Gaopeijiales*, including the novel cultured strains (Fig. 6D and 7; Fig. S9) (7). They could contribute to heme production in natural environments to fulfill the requirements of prevalent heme auxotrophs (59).

#### Secondary metabolite biosynthesis

Secondary metabolites produced by microorganisms affect community members and their environments and are precious sources for the development of antibiotic-active substances (60). The secondary metabolite biosynthesis gene clusters (BGCs) in *Gaopeijiales* genomes/MAGs were analyzed (Fig. S11; Tables S12 and S13). Terpene synthases, polyketide synthases (PKSs), and ranthipeptide (cysteine-rich peptide) synthases ranked as the most frequent BGCs in *Gaopeijiales* (Fig. S11). Ranthipeptide, a radical non-α-carbon thioether peptide, represents a newly described class of ribosomally synthesized and post-translationally modified peptide (RiPP) (61). In addition, the host-associated *Gaopeijiales* exhibited a prominent capacity for secondary metabolic synthesis (Fig. S11). The unspecified ribosomally synthesized and post-translationally modified peptide product (RiPP-like) and proteusin were synthesized predominantly by *Gaopeijiales* members from host environments (Fig. S11). The details of secondary metabolite analysis of the four novel strains in this study are shown in Table S14. They contained multiple BGCs and might synthesize several secondary metabolites, such as ajudazol A, 2,3-dihydroxybenzoylserine, heme D1, N-tetradecanoyl tyrosine, and rhizomide. Ajudazol A, a unique isochromanone derivative exhibiting antifungal activity as an inhibitor of mitochondrial electron transport, was initially isolated from *Chondromyces crocatus* (62–64). *In situ* marine environments, the novel strains might play a significant role in shaping microbial community composition through antagonistic effects. The BGCs responsible for ajudazol A in the four novel strains shared a 53% similarity with the BGCs identified in *C. crocatus* (62). Notably, a gene cluster identical to the known BGC responsible for rhizomides was found in strain Y43 (65). Rhizomides are a family of depsipeptide macrolactones synthesized by a non-ribosomal peptide synthetase (NRPS) and possess antifungal activity (66–68). These observations suggest that *Gaopeijiales* is a promising source of novel bioactive compounds with potential applications.

### Description of the novel taxa

#### Description of *Gaopeijia* gen. nov.

*Gaopeijia* (Gao.pei.ji'a. N.L. fem. n. *Gaopeijia,* named in honor of Pei-Ji Gao, a Chinese Professor and educator, studying the conversion and utilization of renewable lignocellulosic resources by microorganisms). Cells are Gram-stain-negative, rod-shaped, facultatively anaerobic, and non-motile with a chemoorganoheterotrophic metabolism. Cells divide by binary fission. The major fatty acids include iso-C_15:0_, C_16:1_ *ω*5*c*, and iso-C_14:0_. The primary respiratory quinone is MK-6. The major polar lipids are phosphatidylethanolamine, phosphatidylglycerol, diphosphatidylglycerol, and aminolipid. The type species is *Gaopeijia maritima*.

#### Description of *Gaopeijia maritima* sp. nov.

*Gaopeijia maritima* (ma.ri'ti.ma. L. fem. adj. *maritima*, marine) exhibits the following characteristics in addition to the characteristics that describe the genus. Colonies appear translucent, smooth, and circular, and exhibit a pink or salmon color, with diameters ranging from 0.1 to 1 mm. Cells are approximately 0.4–0.6 µm wide and 0.7–4.5 µm long. Growth is observed at temperatures of 20°C–45°C (optimum, 35–37°C), pH 6.5–9.0 (optimum,7.5–8.5), and with 1%–8% (wt/vol, optimum, 3%) NaCl. Growth was observed under aerobic and microaerophilic (8%–9% O_2_) conditions, but not under anaerobic conditions. Tween 20 is hydrolyzed, but Tween 80, starch, cellulose, alginate, casein, and ager are not. The activities of catalase, oxidase, alkaline phosphatase, esterase (C4), esterase lipase (C8), leucine arylamidase, valine arylamidase, cystine arylamidase, α-chymotrypsin, acid phosphatase, and naphthol-AS-BI-phosphohydrolase are positive, while lipase (C14), galactosidase, β-glucuronidase, glucosidase, N-acetyl-β-glucosaminidase, α-mannosidase, and α-fucosidase show negative results. Acids are produced from d-ribose, d-fructose, l-sorbose, d-tagatose, potassium 5-ketogluconate, and esculin ferric citrate. The type strain is DH-78^T^ (=MCCC 1H01409^T^=KCTC 102002^T^) and the reference strains are DH-20 (=MCCC 1H01410 = KCTC 102003), CCK-12 (=MCCC 1H01365 = KCTC 102004), and Y43 (=MCCC 1H01416). The DNA G + C content of type strain is 70.4%.

#### Description of *Gaopeijiaceae* fam. nov.

(Gao.pei.ji.a.ce'ae. N.L. fem. n. *Gaopeijia*, type genus of the family; suff. -*aceae*, ending to denote a family; N.L. fem. pl. n. *Gaopeijiaceae*, family of the genus *Gaopeijia*). The description is the same as for the genus *Gaopeijia*. The type genus is *Gaopeijia* gen. nov.

#### Description of *Gaopeijiales* ord. nov.

(Gao.pei.ji.a'les. N.L. fem. n. *Gaopeijia*, type genus of the order; suff. -*ales*, ending to denote an order; N.L. fem. pl. n. *Gaopeijiales*, order of the genus *Gaopeijia*). The description is the same as for the genus *Gaopeijia*. The type genus is *Gaopeijia* gen. nov.

## MATERIALS AND METHODS

### Enrichment, isolation, and cultivation of *Gemmatimonadota*

The original marine sediment samples were collected from Xiaoshi Island (122°6′20″E, 37°32′30″N) in Weihai, China in October 2022. The temperature of the sediment samples was 20°C, the salinity was 24‰, the pH was 7.3, and the depth was 5–10 cm. Approximately 20 g of sediment was added to a sterilized conical flask containing enrichment medium (per liter of seawater: 0.1 g yeast extract, 0.5 g peptone; pH 7.6), and the mixtures were incubated for 0, 5, 12, 21, and 30 days in a shaker (180 r/min) at 28°C. The aerobic enrichment cultures were diluted with sterilized seawater using the standard 10-fold dilution technique, and 100 µL dilution was spread on 1/10 marine agar 2216 (MA; Becton Dickinson). The plates were then incubated at 28°C for 7 days. Strain colonies were selected from cultures enriched for different periods, and purified by plate streaking using marine agar 2216 (MA; Becton Dickinson). Strains CCK-12, DH-78^T^, DH-20, and Y43 were isolated from 12-, 21-, 21-, and 30-day enriched samples, respectively. Pure cultures were stored at –80°C in a sterile 1% (wt/vol) saline solution supplemented with 20% (vol/vol) glycerol.

### Morphology, phenotype, and chemotaxonomic characterization

To determine the morphological characteristics of the isolates, strains DH-78^T^, DH-20, CCK-12, and Y43 were incubated in marine broth 2216 (MB; Becton Dickinson) at 35°C for 3 days. Cell morphology and size were observed using light microscopy (E600, Nikon), scanning electron microscopy (Nova NanoSEM450, FEI), and transmission electron microscopy (JEM-1200EX, Jeol). The Gram staining reaction was tested employing the method described previously (69). Anaerobic and microaerophilic growth were tested by incubating strains on MA with or without 0.1% (wt/vol) KNO_3_ for 14 days using anaerobic (Hopebio HBYY001) and microaerophilic (Hopebio HBYY008, 8%–9% O_2_) generation bags. Growth ranges and optima of temperature were determined on MA at 0, 4, 15, 20, 25, 28, 30, 33, 35, 37, 40, 43, 45, and 50°C. Growth with different NaCl concentrations (0%−10%, wt/vol, in 1% intervals) was tested in the following medium (0.1% yeast extract, 0.5% peptone), prepared with artificial seawater (per liter: 3.2 g MgSO_4_, 2.2 g MgCl_2_, 1.2 g CaCl_2_, 0.7 g KCl, 0.2 g NaHCO_3_). Moreover, the pH range for growth was determined by adding different pH buffers (20 mM) to the MB medium, with pH values tested ranging from 5.5 to 9.5 (37). Bacterial growth was monitored using a high-throughput real-time microbial growth analysis system (MicroScreen-HT, Jieling Instrument Manufacturing Co., Ltd, Tianjin).

Oxidase activity was determined by employing an oxidase reagent (BioMérieux). The H_2_O_2_ solution (3%, vol/vol) was added to fresh colonies, and bubbles were observed to measure catalase activity. To detect the hydrolytic activity of the four strains, starch (2%, wt/vol), alginate (2%, wt/vol), CM-cellulose (0.5%, wt/vol), casein (1% skimmed milk, wt/vol), and Tween (20, 40, 60, and 80, 1%, wt/vol) were added to the MA medium, respectively (69). The fermentative acid-producing activity and sole carbon source oxidation tests were performed using API 50CH kits (BioMérieux) and Biolog GEN III MicroPlates, respectively. Other physiological and biochemical characterizations were assessed using API ZYM and 20E kits (BioMérieux). All API and Biolog assays were implemented according to the manufacturer’s instructions, except that the NaCl concentration was adjusted to be optimal.

The four strains were cultured in MB under optimum conditions, and the cells harvested from MB at the late stage of the exponential growth phase were freeze-dried for fatty acids, isoprenoid quinone, and polar lipids analyses. For cellular fatty acid analysis, fatty acid methyl esters (FAMEs) were obtained by saponification, methylation, and extraction, and then analyzed using a gas chromatograph (6890N, Agilent) and the Sherlock Microbial Identification System (MIDI, version 6.1) (70). Isoprenoid quinones were extracted from freeze-dried cell material (300 mg) and separated using silica-gel thin-layer chromatography (TLC) plates, as described previously (71). Respiratory quinones were identified using high-performance liquid chromatography (HPLC; LC-20AT, Shimadzu). Polar lipids were extracted using a solution consisting of chloroform, methanol, and water (2.5:5:2, vol/vol/vol), and analyzed using the two-dimensional TLC method (72).

### 16S rRNA gene phylogenetic analysis and BOX-PCR

Genomic DNA was extracted using a bacteria genomic DNA kit (Takara) according to the instructions. The 16S rRNA gene was amplified and sequenced following the methodology described previously (73). Complete 16S rRNA gene sequences were acquired from the individual genome using the ContEst16S algorithm (74). Alignment analysis was performed using the EzBioCloud server (http://www.ezbiocloud.net/) and the NCBI basic local alignment search tool (BLAST) (https://blast.ncbi.nlm.nih.gov/Blast.cgi). The phylogenetic tree was reconstructed preliminarily based on sixty-one 16S rRNA gene sequences using IQ-TREE (version 1.6.12) with the TIM3 + F + I + G4 model (75). The sixty-one 16S rRNA gene sequences include those used to reconstruct the phylogenetic tree in previous work proposing the class *Longimicrobiia* (23). Multiple sequences were aligned using the method MAFFT (76) and then trimmed employing the tool trimAl (77). To further clarify the phylogenetic placement of the novel isolates and reconstruct a non-redundant taxonomic framework of *Gemmatimonadota*, 651 high-quality representative 16S rRNA gene sequences (Table S3, more than 1,200 bp) from cultured strains and uncultured groups were downloaded from EzBioCloud database (version 20230823) (78). These sequences in the database belong to four classes and seven orders, including *Gemmatimonadales* and *Longimicrobiales* (4, 23), with five additional unclassified orders (EU374070, HQ727642, HQ190281, GQ263235, and GU568020). A robust phylogenetic tree was reconstructed using FastTree with default parameters and IQ-TREE with the GTR + F + I + G4 model (75). Bootstrap analysis was performed with 1,000 replications to evaluate tree topologies. Phylogenetic trees were visualized on the Tree Visualization By One Table (vBOT) (79). The subgroup designations were confirmed when one cluster was monophyletic by two phylogenetic trees constructed by different programs using the maximum likelihood approach (80–82).

The BOX element (BOXA1) was amplified using the BOXA1R primer (83). The PCR cycling program was as follows: (i) one cycle of 94°C for 3 min; (ii) 35 cycles of 94°C for 30 s, 52°C for 1 min, 68°C for 8 min; and (iii) one cycle of 68°C for 16 min.

### Genome sequencing, phylogenomics, and genomic analysis

The extraction and purification of genomic DNA was carried out using a bacteria genomic DNA kit (Takara), and the complete genome of strain DH-78^T^ was sequenced by Majorbio Bio-pharm Technology Co., Ltd. (Shanghai, China) employing the Illumina NovaSeq 6000 and PacBio Sequel II platforms. The raw reads were filtered using the data quality control software Fastp (version 0.20.0) (84) and the high-quality clean data were assembled using the hybrid assembly pipeline Unicycler (version 0.4.8) (85). As a final step, the assembly was polished by Pilon (version 1.22) using short-read alignments, reducing the rate of small errors. The draft genomes of strains DH-20, CCK-12, and Y43 were sequenced by Novogene Bioinformatics Technology Co., Ltd. (Beijing, China) employing the Illumina NovaSeq 6000 platform and were assembled using the tool SPAdes (version 3.15.5) (86).

The average amino acid identity (AAI) and the percentage of conserved proteins (POCP) value were calculated using R script (https://github.com/2015qyliang/POCP) with the methods described previously (29, 87). The e-value threshold, percent sequence identity, and percent alignment length parameters were set to 1e-5, 40%, and 70% in the AAI calculation. Genomes affiliated with *Gemmatimonadota* and basic metadata (genome size, GC content, and environmental origin) were obtained from GTDB (release 214.0, https://data.ace.uq.edu.au/public/gtdb/data/releases/release214/214.0/) (88, 89). The genomes were checked for completeness and contamination using CheckM (90). Finally, 429 genomes with >80% completeness and <5% contamination, were chosen for phylogenomic analysis (Table S5). The concatenated alignment sequences of 120 ubiquitous single-copy proteins were obtained using GTDB-Tk (version 2.1.1) (91). The maximum-likelihood phylogenetic tree based on these amino acid sequences was reconstructed using IQ-TREE with the LG + F + I + G4 model (75).

Genome annotations of cultured strains were performed using the NCBI Prokaryotic Genome Annotation Pipeline (PGAP) based on *ab initio* gene prediction algorithms and homology-based methods (92). Clusters of orthologous groups (COGs) analysis was accomplished using eggNOG-mapper (http://eggnog-mapper.embl.de/) (93, 94). To further investigate the metabolic potential of *Gaopeijiales*, the genomes of four novel isolates and 129 genomes/MAGs assigned to the novel taxa identified in phylogenomic analysis were dereplicated using dRep (version 3.5.0) with parameters -pa 0.99 and -sa 0.995 (95). Consequently, 101 genomes/MAGs originating from four distinct environments (marine water/sediment, freshwater/sediment, saline soda water/sediment, and host associated) were considered for subsequent metabolic analysis (Table S5). Functional annotation and metabolic reconstruction of MAGs were performed using METABOLIC (version 4.0) (96) and the Kyoto Encyclopedia of Genes and Genomes (KEGG) database (97). The metabolic pathway analysis of cultured *Gemmatimonadota* strains was accomplished using BlastKOALA server (version 3.0) in KEGG. The secondary metabolite biosynthesis gene clusters (BGCs) in 101 genomes/MAGs were annotated and analyzed employing local antiSMASH 7.0.1 (98). The bubble plot was created in R (version 4.2.0) using the ggplot2 (version 3.5.1) and gridExtra (version 2.3) packages. Other related charts were plotted using Chiplot (https://www.chiplot.online/) and prettified using Adobe Illustrator CC 2018.

### Analysis of environmental distribution

The distribution and diversity of *Gaopeijiales* were assessed using the Integrated Microbial Next-Generation Sequencing database (IMNGS), a platform for global assessment of 16S rRNA gene diversity in a broad range of microbial ecosystems (99). The abovementioned representative sequences affiliated with *Gaopeijiales* were searched using a 97% similarity threshold in a total of 95,549 16S rRNA gene amplicon data sets obtained from different environments. These data sets are available in the Sequence Read Archive (SRA) repository hosted by the International Nucleotide Sequence Database Collaboration (GenBank, DDBJ, and EMBL) server. The relative abundance of *Gaopeijiales* in each data set was determined by calculating the number of hit sequences as a percentage of the total sequence count. Statistically significant differences between the abundance of *Gaopeijiales* in environments, as well as the abundance of each *Gaopeijiales* subgroup across environments, were conducted in R using a pairwise Wilcoxon test. *P*-values were corrected using the Bonferroni correction method (100, 101). The map with the worldwide distribution of *Gaopeijiales* was created in R using the ggplot2 (version 3.5.1) and maps (version 3.4.2) packages. The boxplots showing the relative abundance of *Gaopeijiales* were generated in R using the ggplot2 package.

### High-through sequencing analysis of 16S rRNA gene

For comprehensive analysis of the novel taxon’s presence and relative abundance in the aerobic enrichment system, high-throughput sequencing was applied targeting the V3-V4 variable regions of the 16S rRNA gene. Genomic DNA from aerobic enrichment samples (0-, 5-, 12-, 21-, and 30-day incubation, 4 parallel at each time point, a total of 20 samples) was extracted using commercially available kits and protocols as described previously (26) and was verified *via* agarose gel electrophoresis. The extracted DNA was used to amplify the 16S rRNA gene using the forward primer 338F (5′-ACTCCTACGGGAGGCAGCA-3′) and reverse primer 806R (5′-GGACTACHVGGGTWTCTAAT-3′). As described in a previous study (102), the PCR thermal program and amplicon sequencing were performed by Majorbio Bio-pharm Technology Co., Ltd. (Shanghai, China) based on the Illumina MiSeq PE300 platform. The pair-end (PE) reads were assembled using the tool Flash (version 1.2.11) (103) and then filtered with the data quality control software fastp (version 0.20.0) (84). The operational taxonomic unit (OTU) cluster analysis was conducted using Uparse (version 11) with a threshold of 97% (104). The tool RDP Classifier (version 2.13) (105) and the SILVA database (release 138) (106) were employed to obtain the species classification information corresponding to each OTU representative sequence. OTUs affiliated with *Gemmatimonadota* were manually extracted from the analyzed data, and subsequently, these OTUs representative sequences (Table S8) and the sixty-one 16S rRNA gene sequences (described above) were used to reconstruct the phylogenetic tree. The tree was reconstructed using IQ-TREE with the TIM3 + F + I + G4 model (75).

## Data Availability

The raw amplicon sequencing data have been deposited to NCBI Sequence Read Archive with accession number PRJNA1097817. GenBank accession numbers of 16S rRNA gene sequences and genomes/MAGs can be found in the supplemental material.

## SUPPLEMENTAL MATERIAL

Supplemental figures msystems.00535-24-s0001.pdf Fig. S1 to S11.

Supplemental tables msystems.00535-24-s0002.xlsx Tables S1 to S14.

ASM does not own the copyrights to Supplemental Material that may be linked to, or accessed through, an article. The authors have granted ASM a non-exclusive, world-wide license to publish the Supplemental Material files. Please contact the corresponding author directly for reuse.
