# Supplementary material for: Description of the first marine-isolated member of the under-represented phylum Gemmatimonadota, and the environmental distribution and ecogenomics of Gaopeijiales ord. nov
Source: mSystems. 2024 Nov 19;9(12):e00535-24. doi: 10.1128/msystems.00535-24 (PMC11651109; doi:10.1128/msystems.00535-24)
Supplement: Supplemental figures — Fig. S1 to S11. [file msystems.00535-24-s0001.pdf]

Supplementary Material

Description of the first marine-isolated member of the under-represented phylum *Gemmatimonadota*, and the environmental distribution and ecogenomics of *Gaopeijiales* ord. nov

Yu-Qi Ye[1], Meng-Qi Ye[1,2], Xin-Yue Zhang[3], You-Zhi Huang[4], Zi-Yang Zhou[1], Yan-Jun Feng[3] and Zong-Jun Du[1,2,5,*]

[1]Marine College, Shandong University, Weihai, Shandong, 264209, China

[2]Shandong University-Weihai Research Institute of Industrial Technology, Weihai, Shandong, 264209, China

[3]SDU-ANU Joint Science College, Shandong University, Weihai, Shandong, 264209, China

[4]Shine-Dalgarno Centre for RNA Innovation, Division of Genome Science and Cancer, John Curtin School of Medical Research, Australian National University, Canberra 2601, Australian Capital Territory, Australia

[5]State key Laboratory of Microbial Technology, Shandong University, Qingdao, Shandong, 266237, China

***Correspondence:**

Zong- Jun Du, Email: duzongjun@sdu.edu.cn

Telephone and fax number: +86-631-5688303

This file include:

Supplementary Figures: Fig. S1 to Fig. S11

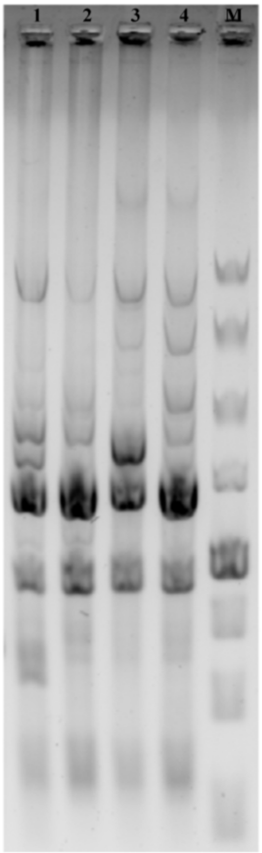

Fig. S1 The BOX-PCR profiles of novel strains *Gaopeijia maritima* DH-78^{T} (lanes 3), DH-20 (lanes 4), CCK-12 (lanes 2), and Y43 (lanes 1). The BOXA1R primer was used. M, 5000-bp DNA size marker.

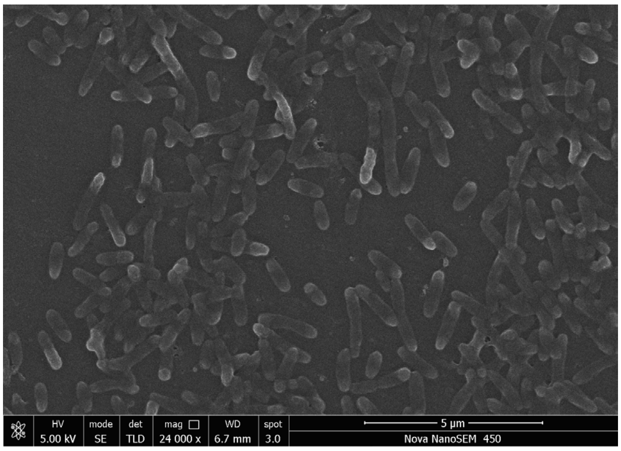

A

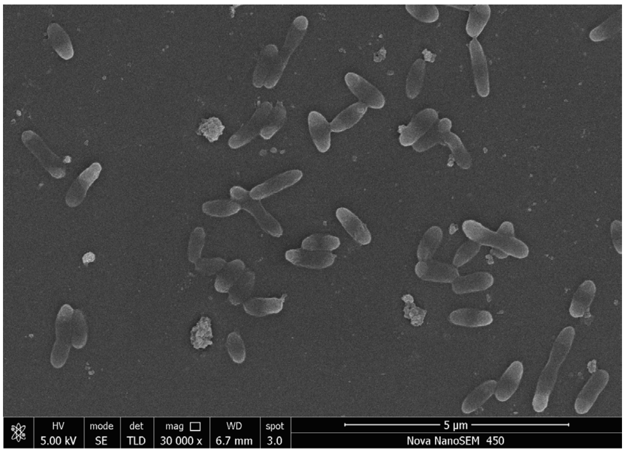

B

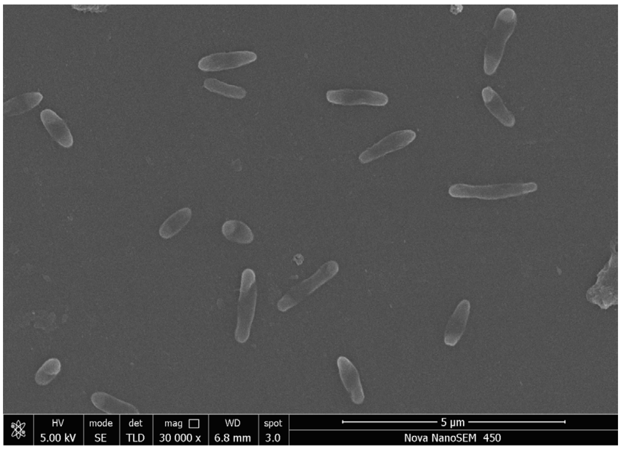

C

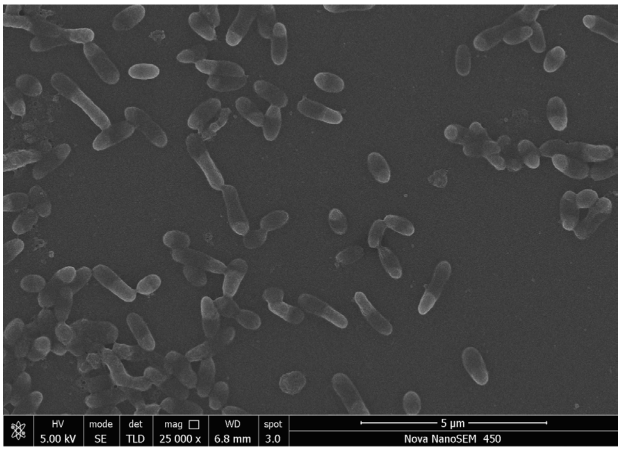

D

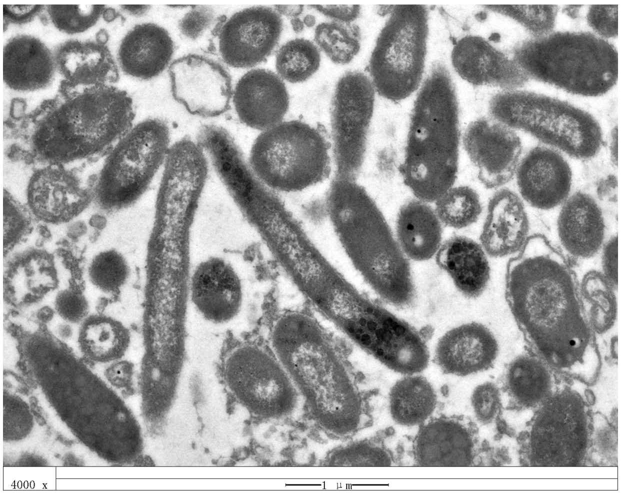

E

Fig. S2 The morphology of *Gaopeijia maritima* cells. Scanning electron micrograph of cells of strains DH-78^T (**A**), DH-20 (**B**), CCK-12 (**C**), and Y43 (**D**). Bar, 5 µm. Transmission electron microscopy of cells of strain DH-78^T (**E**). Bar, 1 µm.

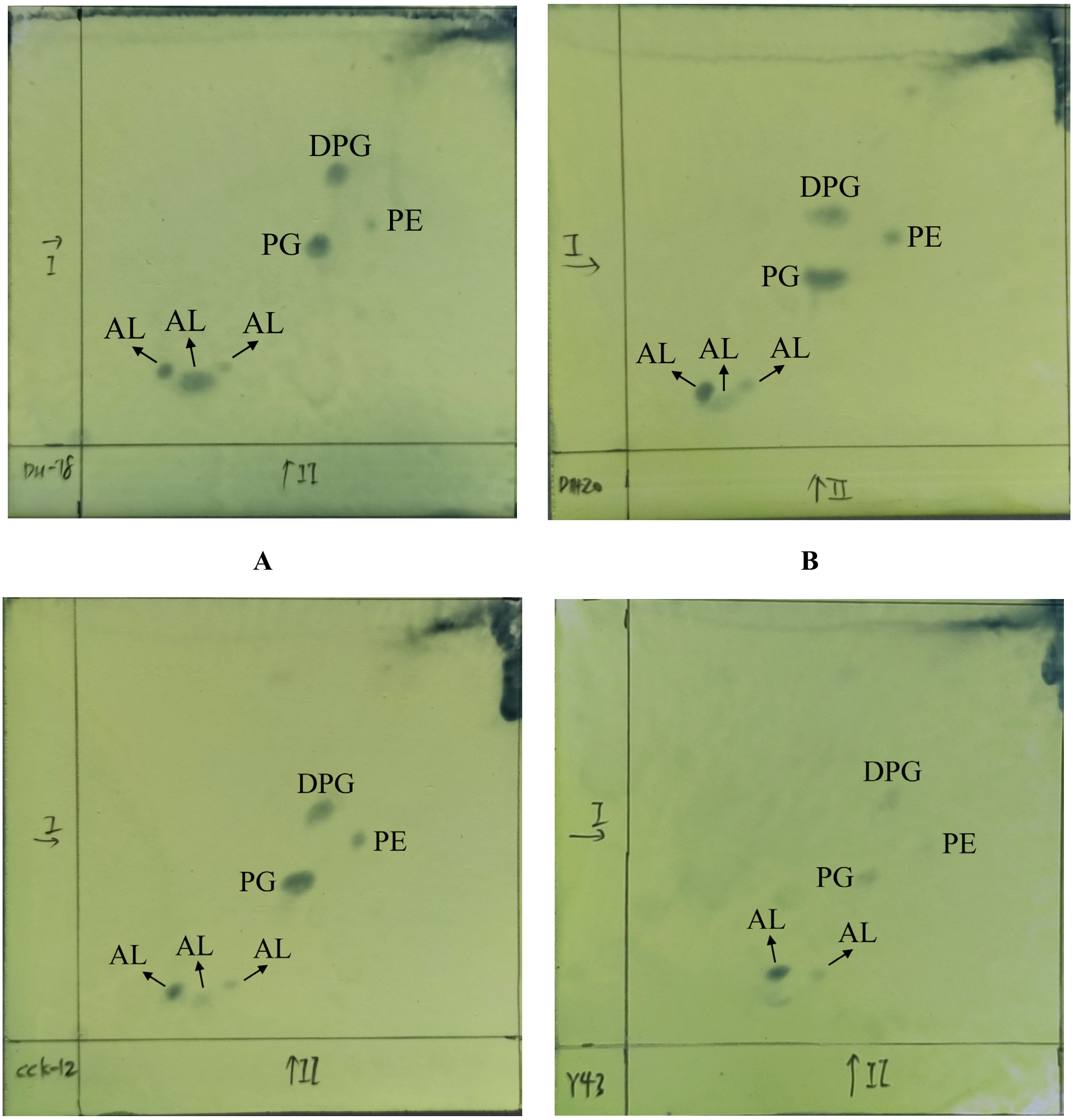

Fig. S3 The two-dimensional TLC plate images of total polar lipids of strains DH-78^T (**A**), DH-20 (**B**), CCK-12 (**C**), and Y43 (**D**). The plate was sprayed with 10% phosphomolybdic acid and heated at 150°C to show all lipids. PE, phosphatidylethanolamine; PG, phosphatidylglycerol; DPG, diphosphatidylglycerol; AL, aminolipid.

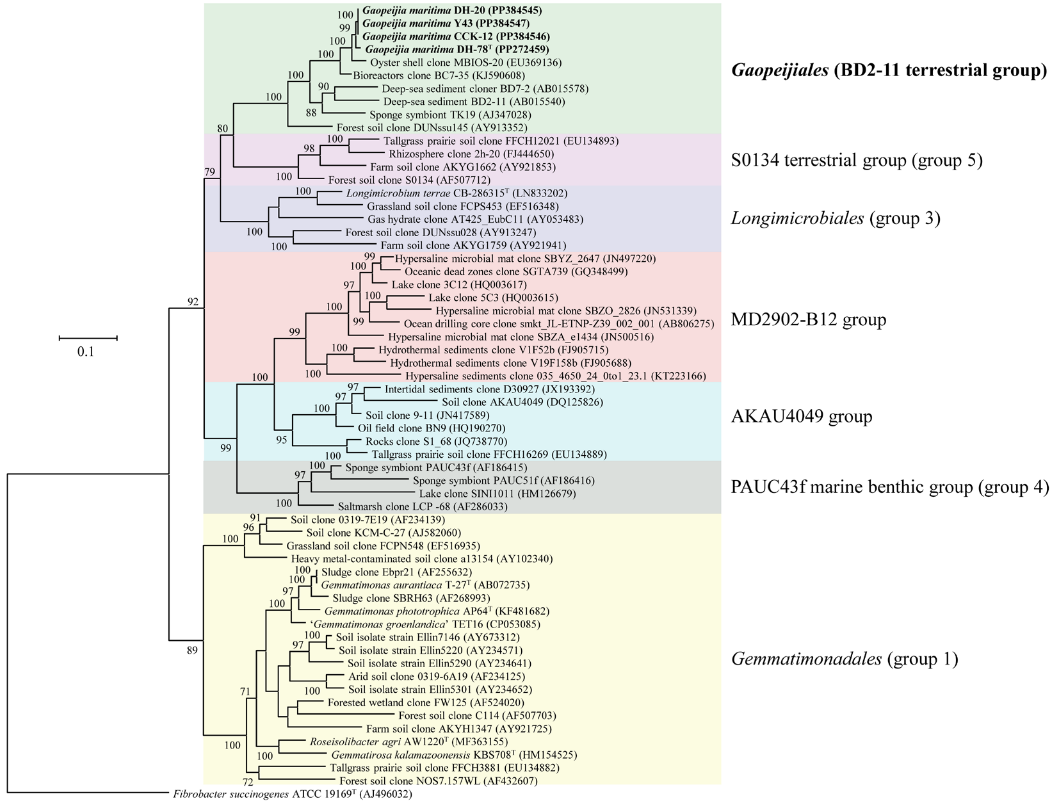

Fig. S4 The maximum-likelihood phylogenetic tree based on 61 *Gemmatimonadota* 16S rRNA gene sequences, including cultured representatives and environmental clones. *Fibrobacter succinogenes* ATCC 19169^T was used as the outgroup. Bootstrap values above 70% (1000 replicates) are shown at branch nodes. Accession numbers are provided in parentheses.

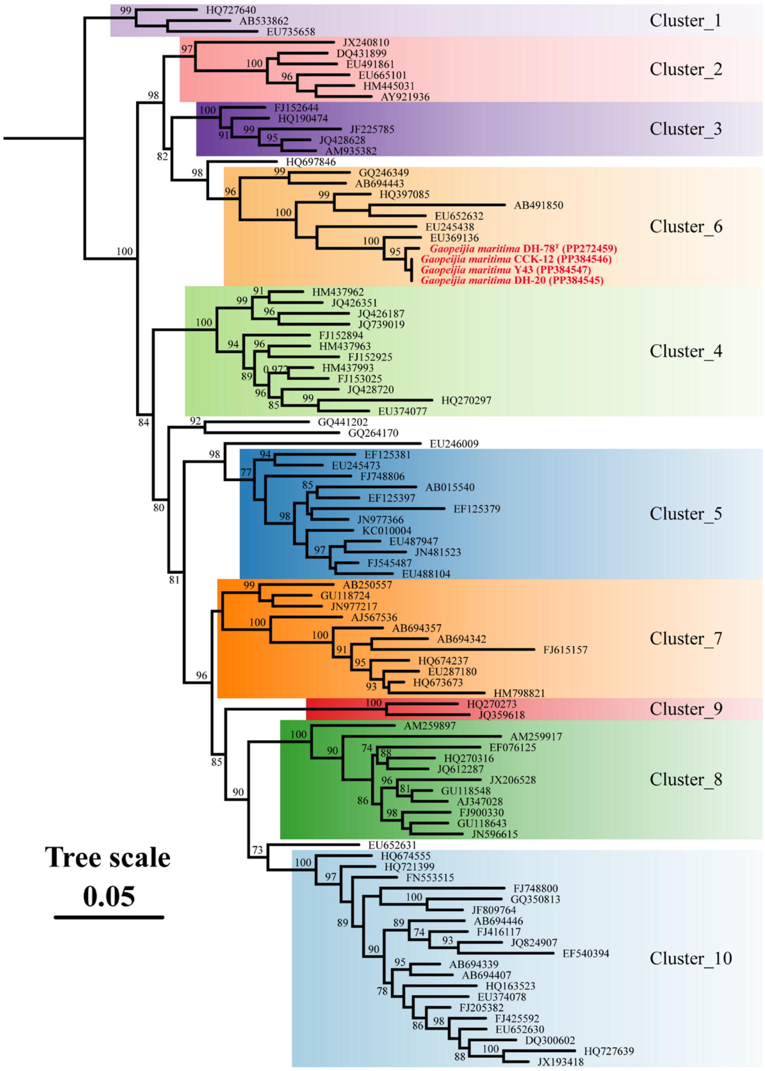

Fig. S5 Phylogenetic tree was reconstructed using FastTree based on 655 high-quality representative 16S rRNA gene sequences and displays only the branches of *Gaopeijiales*. *Fibrobacter intestinalis* NR9^T was used as the outgroup. Bootstrap values above 70% (1000 replicates) are shown at branch nodes. *Gaopeijiales* is divided into 10 subgroups.

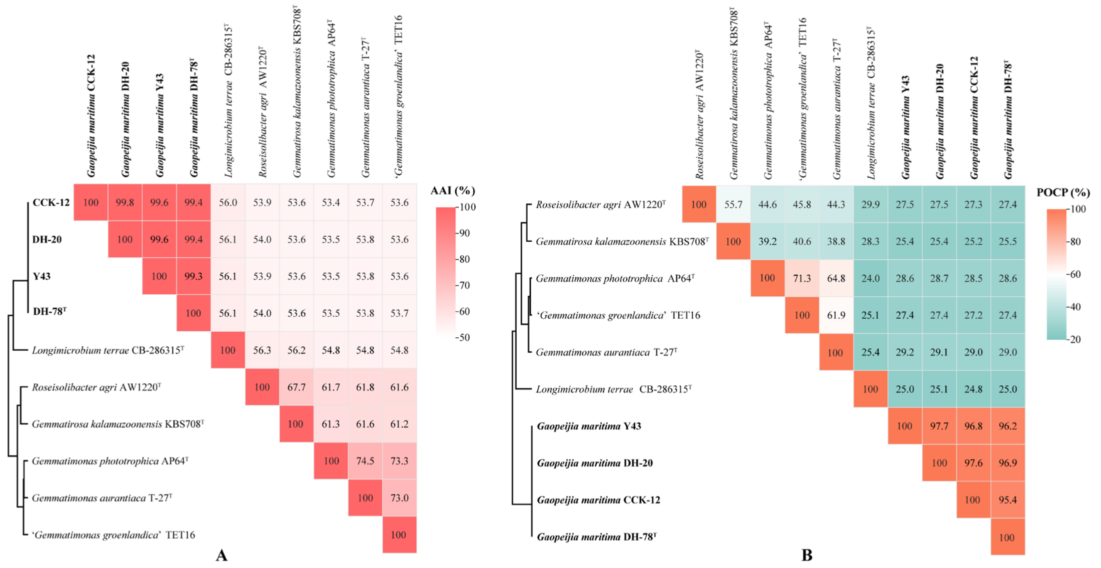

Fig. S6 Heat maps showing AAI and POCP values among DH-78^{T}, DH-20, CCK-12, Y43, and cultured members of *Gemmatimonadota*. **A** Heat map based on AAI values. **B** Heat map based on POCP values. Clustering was performed using the correlation distance method. AAI, average amino acid identity; POCP, the percentage of conserved proteins.

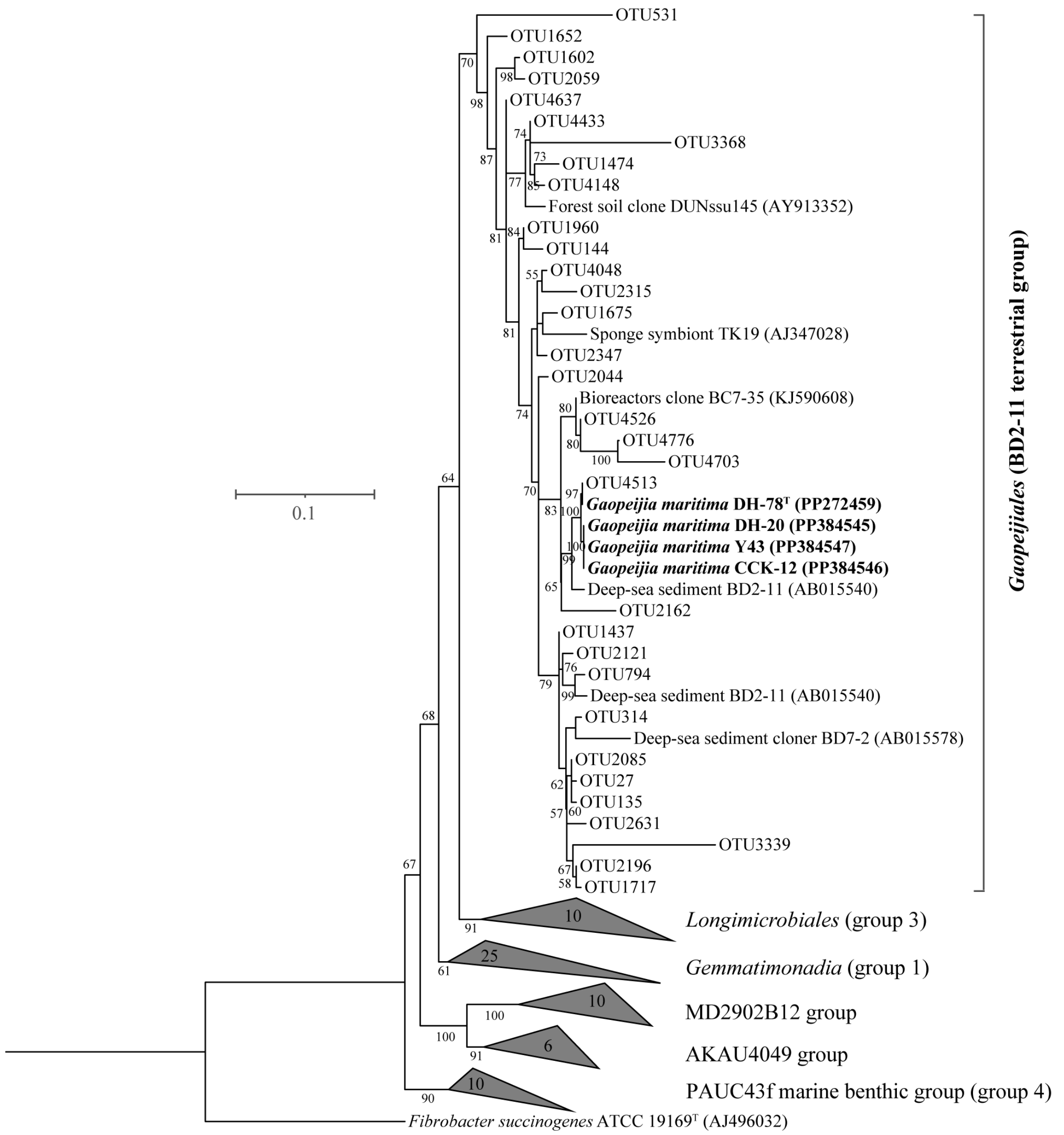

Fig. S7 The maximum-likelihood phylogenetic tree based on 61 *Gemmatimonadota* 16S rRNA gene sequences and 43 OTUs representative 16S rRNA gene sequences affiliated with *Gemmatimonadota*. *Fibrobacter succinogenes* ATCC 19169^{T} was used as the outgroup. Bootstrap values above 50% (1000 replicates) are shown at branch nodes. The details of 43 OTUs representative sequences are shown in Table S8.

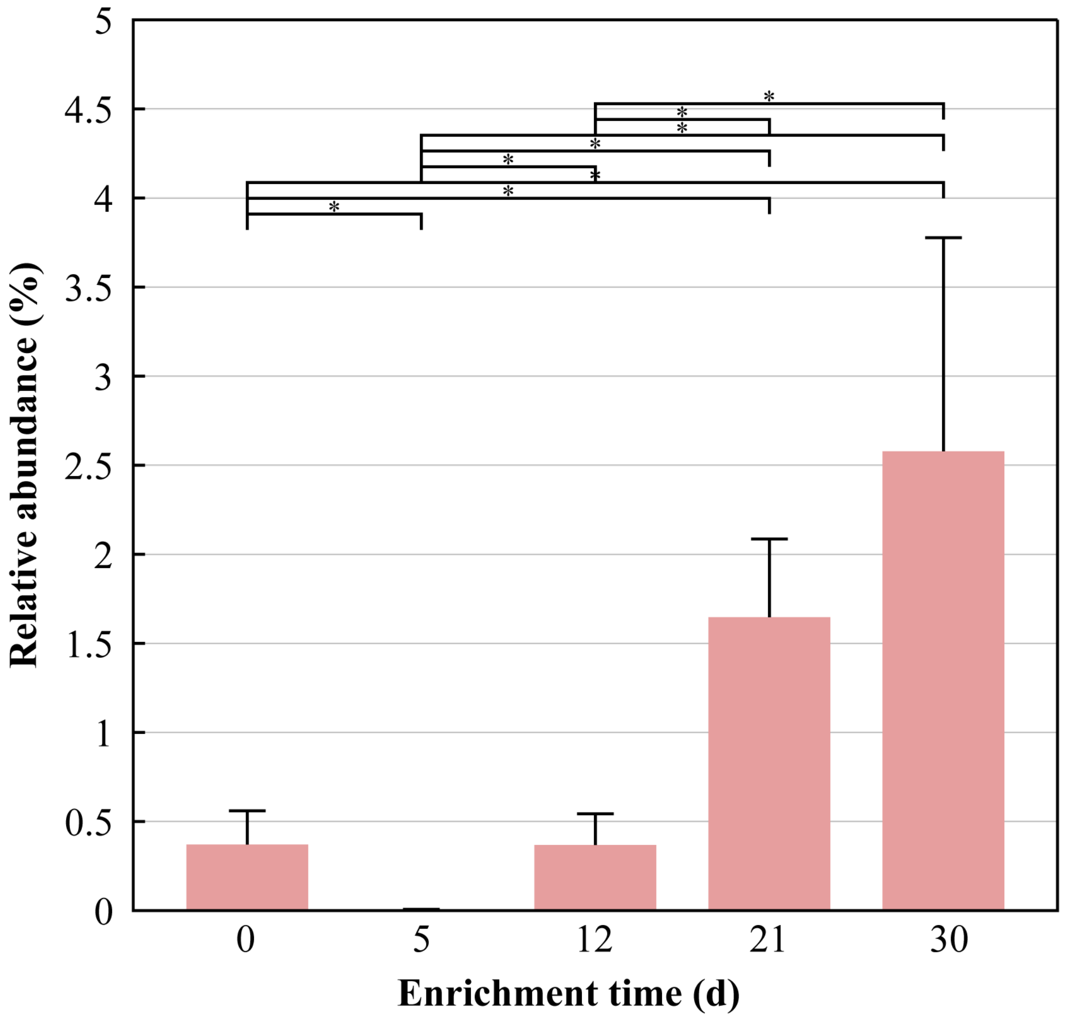

Fig. S8 The relative abundance of *Gaopeijiales* in the enrichment system. The error bar represents the standard deviation. Wilcoxon rank sum test was used to analyze the difference. *, $p < 0.05$.

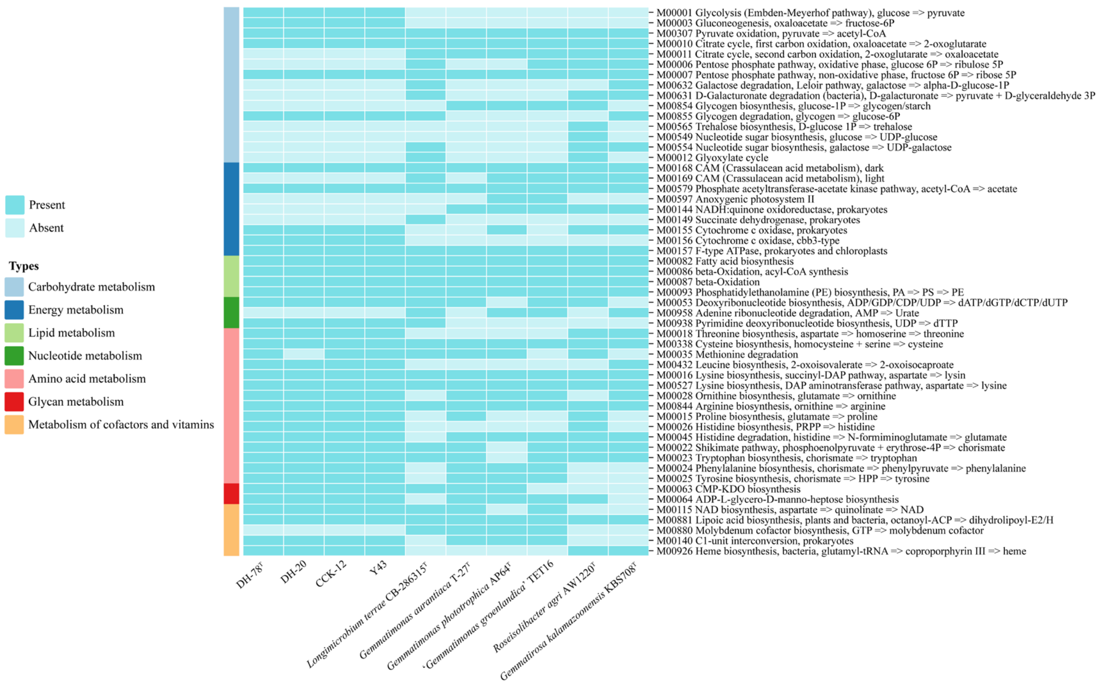

Fig. S9 Heat map of complete and incomplete metabolic pathways in the genomes of strains DH-78^{T}, DH-20, CCK-12, Y43, and cultured members of *Gemmatimonadota*.

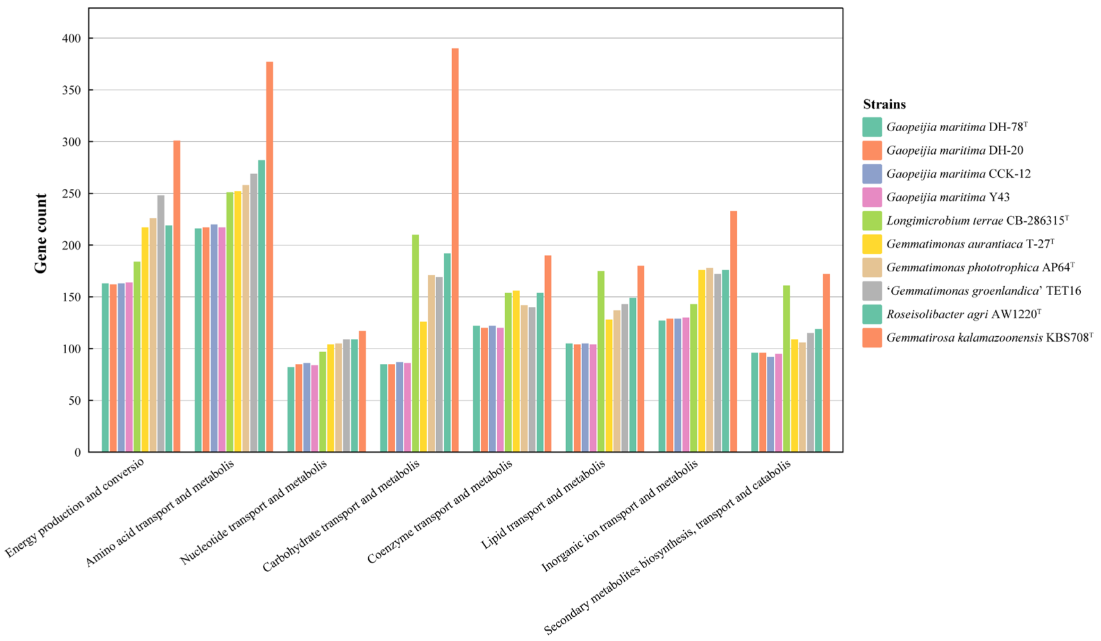

Fig. S10 Gene count of functional categories for strains DH-78^{T}, DH-20, CCK-12, Y43, and cultured members of *Gemmatimonadota*, using eggNOG-mapper.

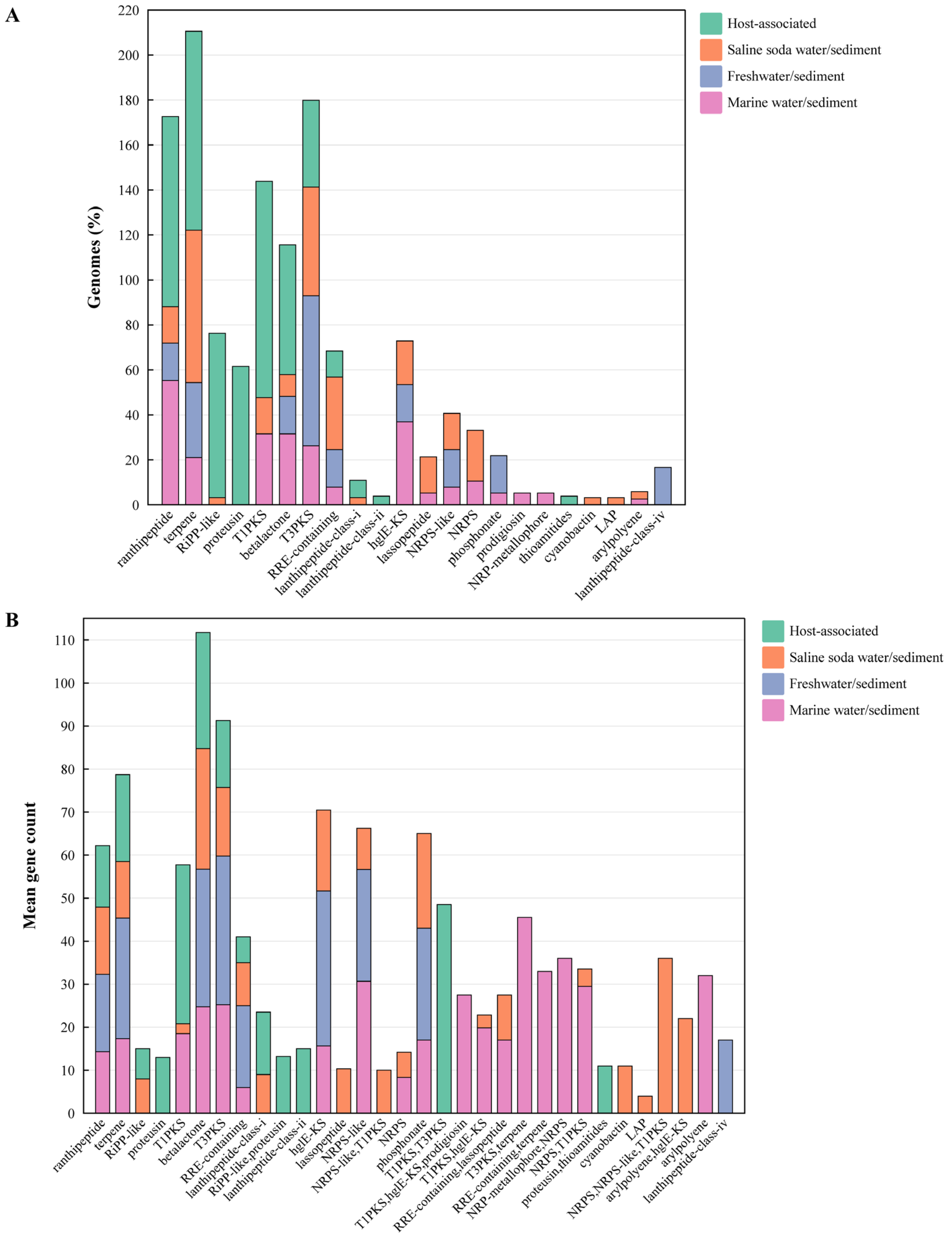

Fig. S11 The secondary metabolite biosynthesis gene clusters (BGCs) in *Gaopeijiales* analyzed using antiSMASH 7.0.1. **A** The percentage of genomes/MAGs that carry BGCs. **B** The mean gene count required to synthesize a class of secondary metabolites. The details are shown in Table S12 and Table S13.
